# Motor Deficits in a Mouse Model of Frontotemporal Dementia: Cortical Tau Pathology and Reduced Nigrostriatal Dopaminergic Markers

**DOI:** 10.3390/cells15201908

**Published:** 2026-10-20

**Authors:** Maciej Zadrożny, Patrycja Zadrożna, Wiktor Niewiadomski, Gernot Riedel, Charles R. Harrington, Claude M. Wischik, Grazyna Niewiadomska, Anna Gasiorowska-Bien

**Affiliations:** 1 Mossakowski Medical Research Institute, 02-106 Warsaw, Poland; mzadrozny@imdik.pan.pl (M.Z.); pdrapich@imdik.pan.pl (P.Z.); wniewiadomski@imdik.pan.pl (W.N.); g.niewiadomska@nencki.edu.pl (G.N.); 2 School of Medicine, Medical Sciences and Nutrition, University of Aberdeen, Foresterhill, Aberdeen AB25 2ZD, UK; c.harrington@abdn.ac.uk (C.R.H.); cmw@taurx.com (C.M.W.); 3 TauRx Therapeutics Ltd., 395 King Street, Aberdeen AB24 5RP, UK; 4 Nencki Institute of Experimental Biology, 02-093 Warsaw, Poland

**Keywords:** frontotemporal dementia, motor cortex, dopaminergic system, tau protein, tauopathy, L66 murine model of FTD

## Abstract

Mice carrying tauopathy-associated mutations are considered good models for frontotemporal dementia because they replicate key features of the human disease. In carriers of the P301S tau mutation, these include degeneration of dopaminergic neurons in the substantia nigra pars compacta (SNpc), reduced striatal dopamine levels, and motor deficits. Such parkinsonian symptoms have been reproduced in mouse models and are accompanied by weakened glutamatergic transmission from the motor and premotor cortex to the striatum. Here, we examined transgenic female homozygous (L66-Homo) and female heterozygous (L66-Het) Line 66 mice, which express the full-length human tau protein carrying the P301S and G335D mutations and NMRI mice as controls. Our focus was on the movement anomalies recorded using the RotaRod and Traction tests, together with histological markers of tauopathy, dopaminergic and glutamatergic systems and possible neurological markers in the motor cortex, striatum and SNpc. Compared to wild-type controls, 9-month-old L66-Homo mice presented with distinct motor impairments in both tests, which were not seen in aged matched L66-Het mice. Compared to L66-Het mice, L66-Homo mice exhibited: (1) more pronounced impairment of the dopaminergic phenotype in SNpc neurons; (2) exacerbated loss of tyrosine hydroxylase staining in striatum; (3) weaker vesicular monoamine transporter 2 staining in SNpc, striatum and frontal cortex; (4) stronger anti-tau staining in striatum and frontal cortex; (5) similar degree of degeneration in frontal cortex; and (6) similar decline in glutaminase staining in striatum and frontal cortex. Surprisingly, no tau-enriched labelling was present in SNpc in both L66-Homo and L66-Het. Therefore, motor impairments in female L66-Homo mice are associated with tau pathology in the striatum and motor cortex, together with a loss of the dopaminergic markers in the SNpc despite an absence of detectable tau immunoreactivity. Altered functional interactions between the striatum and SNpc may contribute to the observed dopamine loss and motor impairment in this mouse model. As only female mice were studied, the applicability of these findings to males remains to be established.

## 1. Introduction

Research has demonstrated a correlation between the variability in normal haplotypes of tau protein and an elevated risk of developing frontotemporal dementia (FTD) syndromes, specifically in progressive supranuclear palsy (PSP) and corticobasal syndrome (CBD), which are categorized as 4R tauopathies [1,2]. The P301S mutation in the human *MAPT* gene is directly associated with familial FTD (FTDP-17). Mice carrying tauopathy-associated mutations such as P301S and P301L are considered good models for frontotemporal dementia because they replicate key features of the human disease. Such mutations result in excessive phosphorylation of tau, thereby mimicking the biochemical changes observed in human FTD [3,4,5]. These support the development of neurofibrillary tangle (NFT)-like aggregates in the cortex, hippocampus, brainstem and spinal cord also in mice, closely resembling human tau pathology [6,7] and, as in FTD patients, these changes progress with ageing [8]. Such mouse models further demonstrate robust glial activation, a hallmark of neurodegeneration in FTD [9,10], and the concomitant aggregation of tau and inflammation results in widespread neuronal death, thereby recapitulating human disease mechanisms [11]. P301S mouse models also demonstrate impaired learning and memory (e.g., open-field water maze performance, deficits in hippocampal long-term potentiation, LTP), and progressive motor impairments. Deficits correlate with spinal cord pathology and tau burden, inducing parkinsonian-like symptoms that are frequently observed in FTD [12]. Consequently, P301S tauopathy mouse models are considered powerful tools in research, since they mirror the genetic, pathological and behavioral hallmarks of human FTD.

Tau transgenic mice carrying the P301S mutation (and G335D to a lesser extent) exhibit impairments to the nigrostriatal dopaminergic system, including degeneration of neurons in the substantia nigra, reduced striatal dopamine levels, and motor deficits. This makes them models for FTD but also for the study of tau-related parkinsonian syndromes [13,14]. The selective vulnerability of nigrostriatal dopaminergic neurons as the underlying cause of the progressive neurodegeneration is characteristic of Parkinson’s disease and the depletion of dopamine is related to the accumulation of both α-synuclein and phosphorylated tau aggregates [15]. Nigrostriatal dopaminergic neurons possess extensive axonal arborizations, elevated metabolic and bioenergetic requirements, and continuous autonomous pacemaking; these properties chronically elevate oxidative burden, disrupt mitochondrial dynamics and mitophagy, and amplify intracellular calcium transients, converging to impair proteostasis and render the cells particularly vulnerable to oxidative damage, mitochondrial failure, and excitotoxic injury [16,17,18]. The interplay between α-synuclein and tau aggregation further accelerates neuroinflammatory responses and neurodegeneration [19,20].

The basal ganglia are recognized as a circular network, through which information from the cerebral cortex descends to the striatum, traverses the direct and indirect pathways that contain segregated D1 and D2 receptors, and returns via the thalamus to the originating cortical region [21,22,23]. Degenerating nigrostriatal projections indirectly affect the functioning of the entire corticostriatal system and have a major impact on the physiology of cortical signals for the integration of sensory information for the generation of movement [24,25,26]. These have been mimicked in mouse models using 1-methyl-4-phenyl-1,2,3,6-tetrahydropyridine (MPTP), among others, with a loss of corticostriatal tone and disturbances in the corticothalamic pathway [27,28,29]. In the MPTP model, reduced synaptic density and weakened glutamatergic transmission are observed in the afferent connections from both motor and premotor cortex to the striatum. All these aspects show enriched tau phosphorylation in PD patients [15]. Previous pharmacological and brain dialysis studies have confirmed that increases in extracellular dopamine levels result in increased phosphorylation of tau in the prefrontal cortex, striatum and hippocampus in mutant P-301L tau mice [30,31] and could conceivably explain the transdiagnostic relevance of psychotic syndromes (schizophrenia, apathy, anhedonia) in some of these models [32,33,34]. These include Line66 (L66) mice, who present with a nest-building and sucrose preference deficit, which could be symptoms of reduced dopamine signaling.

Many mutations in tau protein are associated with frontotemporal dementia, including P301S and P301L [35]. In this study we have examined L66 mice that express the full-length human tau protein, which carries both P301S and G335D mutations under control of the neuron-specific murine Thy-1 promoter [11]. The resulting phenotype involves motor impairments that worsen with age. These manifestations are associated with the progressive accumulation of tau aggregates in various brain regions, closely mirroring the clinical and pathological features of frontotemporal dementia in humans [11]. We have examined the motor behavior of homozygous and heterozygous L66 mice, focusing on the nigrostriatal dopaminergic system and its relationship with cortico-striatal projections to investigate the putative connections between tau, dopamine and movement-related phenotypes.

## 2. Methods

### 2.1. Animals

All animal experiments were conducted with ethical approval (No. WAW2/116/2020) of the Warsaw Local Institutional Animal Care and Use Committee (IACUC) in strict accordance with the European Communities Council Directive (63/2010/EU) and compliant with the ARRIVE guidelines 2.0 [36]. Mice were bred commercially in positive-pressure isolators (Charles River, Margate, UK) and delivered to the local facilities via air at 6 months of age. NMRI wild-type and L66-homozygous (L66^+/+^ − L66-Homo) and heterozygous (L66^+/−^ − L66-Het) female mice were selected (NMRI n = 15; L66-Homo, n = 15; L66-Het, n = 15) in line with our previous work [11]. L66 expresses full-length human tau (amino acids 1–441), which carries the P301S and G335D mutations driven by the neuron-specific murine Thy-1 promoter [11]. Animals were maintained in small groups of 3–5 in open housing in standard Perspex colony cages with wood shavings as bedding (Chipsi, JRS, Rosenberg, Germany) and cotton fiber pads and wood wool nesting material as enrichment until aged 9 months, when experiments proceeded. The facility was run under a 12-h light/dark cycle (lights on at 8 am) at a temperature of 21 ± 1 °C and constant humidity of 50–65%. Animals had *ad libitum* access to water and food pellets (Labofeed B, Wytwórnia Pasz Morawskie, Żurawia, Poland).

The genetic makeup of L66 mice is reminiscent of FTD_tau_ with parkinsonism [37,38] and they have been extensively characterized in terms of tau neuropathology, synaptic activity and protein content, inflammatory profiles, proteomic differences, behavioral phenotypes and glutamatergic and cholinergic transmitter characteristics [11,39,40,41,42].

### 2.2. Protocol of Behavioral Tests

**Figure 1:**
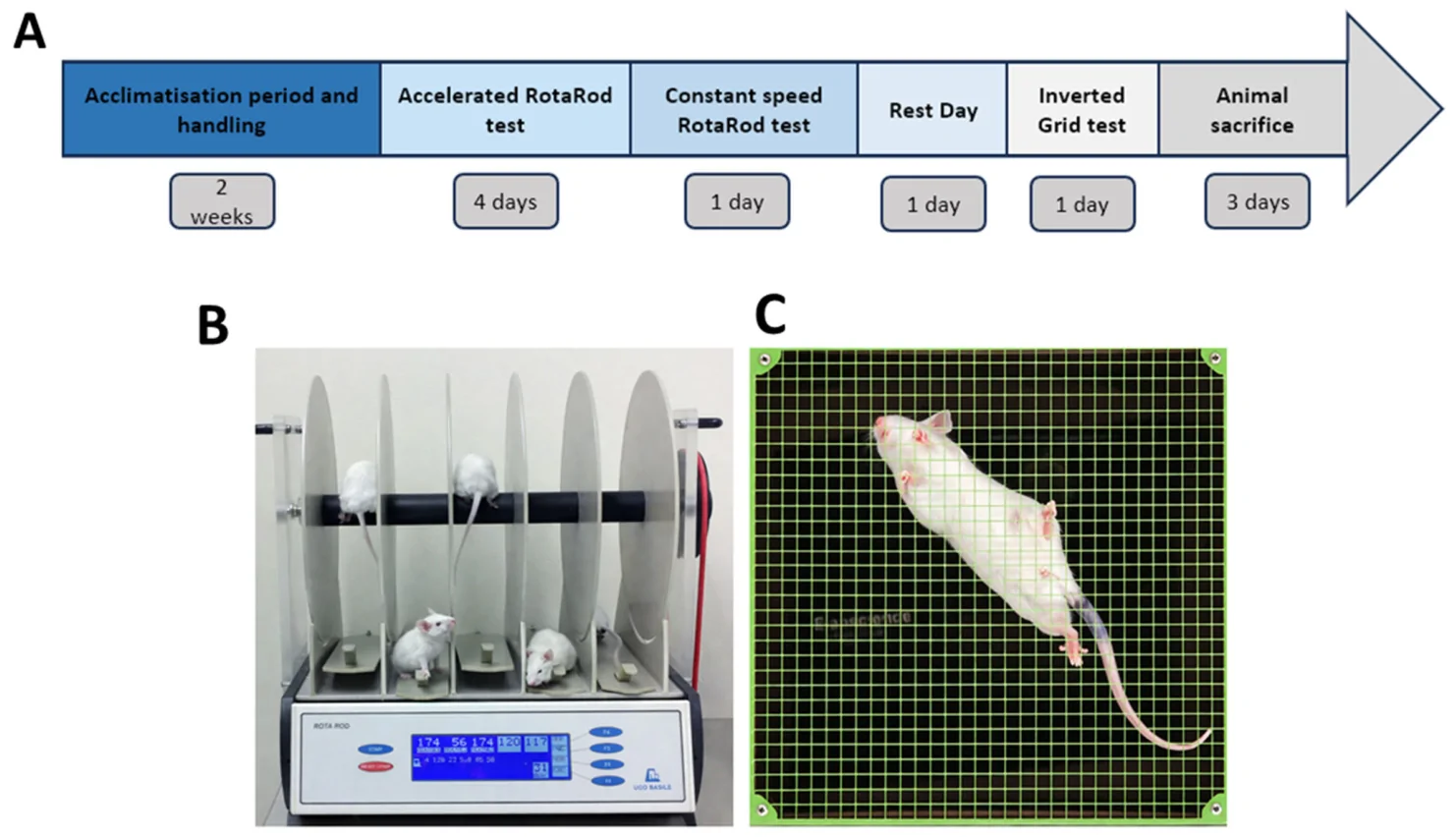
(**A**) Timeline of the experimental procedure, including acclimatization and handling, behavioral testing (accelerated RotaRod, constant speed RotaRod, and inverted grid test), and animal sacrifice. (**B**) Mice performing on a RotaRod to assess motor coordination and balance. (**C**) Top view of a mouse clinging to the underside of the meshed wires after the grid was rotated in the Traction test.

Figure 1 outlines the experimental procedures and time course. All experiments were conducted in quiet experimental rooms with the same environmental parameters as the holding rooms. Animals were removed from their holding cages and brought to the experimental room in transport cages (500 cm^3^, polysulfone) with wood shavings as bedding and *ad libitum* water. They were acclimatized to the experimental room for 30 min per day before the experiments were started. After two weeks, two different RotaRod experiments were performed, one with acceleration of the rod (4 trials per day, inter-trial interval (ITI) 2 min), and the second one at a constant speed (5 trials per day, ITI = 2 min). After some resting, a test with the inverted grid (Traction test) (3 trials, ITI = 1min) was administered and this was followed on subsequent days by tissue harvest. All experiments were performed during the light cycle between 10am and 4pm and animals were returned to their holding cages upon completion of their sessions. All apparatuses were cleaned with Velox Top AF (Medisept, Lublin, Poland) between animals.

#### 2.2.1. RotaRod Tests

A four-lane RotaRod for rats (Model 47700 ROTAROD, Ugo Basile, Comerio, Italy) was utilized in the study of motor learning and performance. Two tests were performed: firstly, a test with accelerating rotations and secondly, a test using constant rod rotation speeds. At the commencement of each trial, the mouse was positioned on a stationary rod with its body perpendicular to the axis of rotation. Once four mice were loaded into the apparatus (Figure 1B) in Exp. 1 (accelerated test), rotation of the rod was increased from 0 to 45 rotations per minute (rpm) over a period of 300 s. The trial ended either when the mouse fell off the rod or when it managed to stay on it for a period of 300 s. The performance of the mice was measured as the duration (in seconds) that they remained on the rotating rod, which is equated to the rpm sustained. Motor coordination was recorded as performance on day 1; all other days/trials assessed motor learning. In Exp. 2 (constant speed test), mice were loaded onto the lanes and the RotaRod was accelerated to and maintained at a constant speed. In five trials, the following velocities were applied for 1 min each: 5, 15, 25, 35 and 45 rpm. Again, performance was measured in terms of the time (in seconds) spent on the rod at a given speed of rotation. Allocation to the lanes was counterbalanced for genotype and trial and experimenters were blinded to genotype during testing. No power calculations were performed *a priori*.

#### 2.2.2. Traction Test

A box with a 12 × 12 cm green wire mesh floor, which contained 5 × 5 mm holes, surrounded by opaque walls measuring 9 cm in height, was utilized for the Traction test. The mouse was placed at the center of the mesh, after which the box was inverted. This resulted in the mouse becoming suspended on the wires and clinging to the grid (Figure 1C). The grid was 24 cm above the floor, and soft padding was placed underneath it to prevent injury from falling. Mice possess a natural ability to either remain stationary on the grid or to move freely around it in this hanging position. Each mouse performed three trials, with a one-minute ITI. A trial ended either when a mouse fell off the grid or when a max. time of 30 s had elapsed [29]. The trial duration of 30 s was chosen based on our previous work as it was attainable for most mice. At the end of the last trial, animals were returned to their home cages and the equipment cleaned with Velox Top AF (Medisept, Lublin, Poland). Trials were recorded by a Sony α37 camera positioned 70 cm above the grid and stored as Moving Picture Experts Group (MPEG) files for offline analysis. The longest trial out of three performed by each animal was selected for analysis in order to obtain a considerable number of Steps and Holds. Time spent clinging to the grid was termed ‘Cling Time’ and is given in seconds. Videos were imported into MATLAB R2022a (The MathWorks, Inc., Natick, MA, USA) and analyzed frame by frame by a rater, who was unaware of the animal’s genotype, using TracMouse [29].

The rater’s task was to identify and tag each individual paw—front left (FL), front right (FR), hind left (HL), and hind right (HR)—along with its position on the grid during instances of *Hold*. The term Hold refers to a state in which a paw remains in a static position and is characterized by the identity of the paw, its location on the grid, and the timestamps marking the onset (*t₍b₎*) and termination (*t₍e₎*) of the Hold. The primary data collected by the rater comprised annotated information detailing every Hold event for each paw. To convert paw positions from pixel units to millimeters, calibration was conducted using the external reference frame of the grid (12 × 12 cm). The movement of the animal was registered in *Steps*. The Step constituted a paw displacement from the initial Hold position (x_i_, y_i_) to the next Hold position (x_i+1_, y_i+1_). The temporal elements of each Step were ascertained as Step duration and Step length. Duration was defined as the temporal interval between the commencement of the Hold to the Step (*t₍e₎*_i_) and the termination time of the next Hold (*t₍b₎*)_i+1_. Step length is defined as the distance between paw positions during a given Hold, and paw positions at the next Hold. These parameters were cumulatively summed by trial and animal, and details are provided in Table S1 (supplementary information).

The following proxies were calculated for each animal: the number of Steps performed by each paw during the trial (Total Number of Steps); the sum of the durations of all Steps during the trial (Overall Duration of Locomotion); the distance covered by each paw during the trial in millimeters (Cumulative Paw Step Distance); the average duration of Steps in seconds (Mean Step Duration); the average length of Steps performed by each paw in millimeters (Mean Paw Step Length); and the average velocity of Steps performed by each paw in millimeters per second (Mean Paw Step Velocity).

Parameters depending on ‘Cling Time’ were normalized to a 30-s trial length. For some animals, even the longest Cling Time out of three trials was less than 30 s; in others, it was a few seconds longer because, once the individual Cling Time exceeded 30 s, a few seconds were needed to terminate the trial. Since fewer steps can be performed in shorter trials, we multiplied these with a correction factor (30 s/Cling time). This would normalize the proxy for all subjects and thus be comparable. Other parameters, i.e., Overall Duration of Locomotion and Cumulative Paw Step Distance, were corrected similarly. For proxies such as Mean Step Duration, Mean Paw Step Length, and Mean Paw Step Velocity, no normalization was required.

### 2.3. Euthanasia of Animals and Tissue Harvesting

The animals were assigned to two histological processing groups (paraffin and frozen sections) based on their performance on day 4 of the Accelerated RotaRod test; they were ranked from the best to the worst within each group and allocated using an alternating procedure.

#### 2.3.1. Transcardial Perfusion and Tissue Collection for Immunohistochemical Analysis of Frozen Sections

The animals were sedated with a mixture of ketamine (100 mg/mL; 75 mg/kg; intraperitoneally) and medetomidine (1 mg/mL; 1 mg/kg; intraperitoneally) in 0.9% NaCl as suggested in Annex IV (Methods of killing animals) of Directive 2010/63/EU of the European Parliament and of the Council from 22 September 2010 on the protection of animals used for scientific purposes. Once fully anaesthetized, animals were placed in a supine position, the chest was opened and a cannula was inserted into the left ventricle of the heart and the right atrium was opened. Cold saline containing heparin (perfusion rate: 30 mL/min) was administered, followed by a fixative of 4% paraformaldehyde with saturated picric acid (perfusion rate: 50 mL/min) and a 5% glycerol solution with 2% DMSO (perfusion rate: 30 mL/min). Once perfusion was complete, the animal was decapitated and the brain removed from the skull.

After extraction, the brains for free-floating sections were placed in paraformaldehyde with picric acid for approximately 24 h at 4 °C. Then they were transferred to 10% glycerol (with 2% DMSO) for another 24h in 4 °C, and next, to 20% glycerol (with 2% DMSO) for 24 h at 4 °C. On the fourth day, the brains were frozen and stored at −80 °C.

#### 2.3.2. Transcardial Perfusion and the Collection of Tissue Samples for Immunohistochemical Analysis of Paraffin-Embedded Sections

Animals were treated as above for tissue harvest, but 4% formaldehyde (perfusion rate: 50 mL over 1.5 min) was used as fixative. Once perfusion was complete, animals were decapitated and brains removed from the skull. The brains were then placed in 10% neutral buffered formalin for two weeks. They were then processed into a series of ethanol solutions of increasing concentration (50% to 100%) until pure, water-free alcohol was reached. The tissue was processed in xylene three times and then infiltrated with histological wax twice. The brains embedded in paraffin blocks were stored at room temperature until sectioning.

#### 2.3.3. Immunohistochemistry Protocol for Free-Floating Sections

Mouse brains were placed on dry ice and cooled in a Leica CM1850 cryostat (Nussloch, Germany) to −27 °C for 45 min. The frozen brains were secured to the cryostat table using a tissue-freezing medium (Cryomatrix™, Thermo Scientific, Rugby, UK) and sectioned coronally at a thickness of 40 μm. Using the mouse brain atlas (Paxinos and Franklin, 2013) as a reference [43], six stereotactic levels were defined within the motor cortex/striatum, spanning 1.54 to −0.02 mm relative to Bregma, and six levels were defined within the midbrain, spanning −2.70 to −3.80 mm relative to Bregma. At each predefined stereotactic level, six consecutive sections were collected at 40 µm from cryostat sections encompassing the motor cortex/striatum and four consecutive sections from the midbrain and distributed over six slides. Thus, each slide contained six sections representing the six stereotactic levels of the motor cortex/striatum and midbrain. This generated 36 sections on six slides for the motor cortex/striatum and 24 sections on six slides for the midbrain, totaling 60 sections distributed across 12 slides. This organization enabled a systematic immunohistochemical analysis of defined rostro-caudal levels using different staining protocols (Supplementary Figure S2). One set from these series was processed for tyrosine hydroxylase (TH) immunoreactivity, one for vesicular monoamine transporter 2 (VMAT-2) and a third one for tau. Free-floating sections were subjected to a rinsing process comprising three cycles of 5 min with 0.1 M phosphate-buffered saline (PBS). Sections were incubated with 1% hydrogen peroxide (H_2_O_2_) in 0.1 M PBS to block endogenous peroxidases, rinsed thrice with 0.1 M PBS and kept for one hour at room temperature with 5% normal goat serum (Vector Laboratories, Cat. No. S-1000-20, Newark, CA, USA) in 0.1 M PBS with 0.3% Triton X-100. Next, samples were incubated with primary antibodies in 5% normal goat serum (NGS), 1% bovine serum albumin (BSA), and 0.3% TritonX100 in 0.1 M PBS at room temperature for one hour before being transferred and kept at 4 °C overnight with continuous agitation. The following primary antibodies were utilized in the study (see Table 1): anti-TH (Merck, Cat. No. AB152, Taufkirchen, Germany) at a 1:1000 dilution, anti-VMAT-2 (Immunostar, Cat. No. 20042, Hudson, WI, USA) at a 1:500 dilution, anti-tau 7/51 antibody (TauRx Therapeutics Ltd., Singapore) at a 1:500 dilution and anti-tau AT8 (Thermofisher Scientific, Cat. No. MN1020, Rockford, IL, USA) at a dilution of 1:200. After washing the sections three times with 0.1 M PBS, they were incubated for one hour at room temperature (RT) with a secondary antibody. For TH and VMAT-2, the secondary antibody was goat anti-rabbit IgG antibody conjugated with horseradish peroxidase (HRP) (Sigma-Aldrich, Cat. No. AP307P, St. Louis, MO, USA) in 5% normal goat serum (NGS), 1% BSA and 0.3% Triton X-100 in 0.1 PBS. For 7/51 and AT8, goat anti-mouse IgG antibody conjugated with HRP (5% NRS, 1% BSA, 0.3% Triton X100 in 0.1 M PBS) was used. The sections were then rinsed three times with 0.1 M PBS. Visualization commenced in a solution of PBS containing 3,3′-diaminobenzidine tetrahydrochloride (DAB, Sigma-Aldrich, St. Louis, MO, USA), H_2_O_2_ and nickel sulfate (NiSO_4_) at concentrations of 0.025%, 0.0125% and 0.04%, respectively. Finally, sections were mounted, air-dried and cover-slipped. Controls for the immunohistochemical procedure were obtained by omitting the primary or secondary antibodies from some slides throughout the procedure. No specific staining was observed for any of the control slides. Total number of slices analyzed differed due to quality of tissue after the reaction. Damaged slices were excluded from the analyses.

#### 2.3.4. Immunohistochemistry Protocol for Paraffin-Embedded Sections

Mouse brains obtained at autopsy were embedded in paraffin wax and cut into 6 µm-thick coronal sections using a microtome (SLEE Microtome CUT5062, Nieder-Olm, Germany). Using the mouse brain atlas (Paxinos and Franklin, 2008) as a reference [43], stereotactic coordinates were determined for the motor cortex (from 1.54 mm to −0.02 mm relative to Bregma) and the midbrain (from −2.70 mm to −3.64 mm relative to Bregma) in sagittal sections of the brain. Within the motor cortex, eight levels separated by equal distances were defined, while six levels were defined in the hippocampus. Tissue was cut into 6 µm-thick sections using a microtome (SLEE CUT, Nieder-Olm, Germany), yielding 20 consecutive sections for each of the defined stereotactic levels. Each of the 20 sections obtained was mounted on a separate microscope slide. Subsequently, sections from consecutive stereotactic levels were serially mounted on the same slides, separately for the motor cortex and the hippocampus. This mounting method made it possible to obtain a set of slides representing the entire cross-section of a given anatomical area (Supplementary Figure S3). The sections were deparaffinized in xylene and a 1:1 solution of xylene and ethanol, then rehydrated in a series of consecutive ethanol solutions with decreasing ethanol concentrations (from 100% to 50%). Antigen exposure was performed in a milky citrate buffer solution (pH 6.0) which was heated to 95–98 °C for 30 min. This was followed by a 15-min cooling period and treatment with 3% hydrogen peroxide in methanol at room temperature for 15 min. The tissue was then rinsed twice in distilled water and once in 0.1 M PBS. It was incubated overnight at 4 °C in a solution containing S-1D12 primary anti-tau antibody (TauRx Therapeutics Ltd., Aberdeen, U.K.), NeuN antibody (Merck, Cat. No. MAB377, St. Louis, MO, USA) or anti-glutaminase (Thermo Fisher Scientific, Cat. No. PA5-35365, St. Louis, MO, USA), and 3% bovine serum albumin (BSA) in 0.1 M PBS. The concentration of each primary antibody is provided in Table 1. The next day, sections were washed three times for five minutes in 0.1 M PBST and incubated with a horseradish peroxidase (HRP)-conjugated goat anti-mouse IgG secondary antibody (Merck, Cat. No. AP307P, St. Louis, MO, USA) for NeuN and S1D12, or with a horseradish peroxidase (HRP)-conjugated goat anti-rabbit IgG secondary antibody (Merck, Cat. No. AP307P, St. Louis, MO, USA) for anti-glutaminase staining, in 5% antibody-appropriate normal goat serum and 1% BSA in 0.1 M PBST, for one hour at room temperature. The sections were then washed three times for five minutes in 0.1 M PBS. Antibody binding was visualized using a solution of diaminobenzidine (DAB, Sigma-Aldrich, USA), hydrogen peroxide and nickel sulfate at concentrations of 0.025%, 0.0125% and 0.04%, respectively, in 0.1 M PBS. The reaction was quenched by adding an excess of 0.1 M PB. Sections were mounted using DePeX (Serva, Heidelberg, Germany), after which the drainage step was performed. Controls for the immunohistochemical procedure were included by omitting the primary or secondary antibodies from some sections throughout the procedure. No specific staining was observed for any of these control sections.

#### 2.3.5. Nissl Staining Protocol for Paraffin-Embedded Sections

The sections were deparaffinized in xylene and rehydrated using a series of consecutive ethanol solutions with decreasing concentrations (from 100% to 50%). After rinsing with distilled water, the sections were stained with acidified cresyl violet (10% acetic acid) for 15 min. After rinsing in distilled water, the differentiation process was performed in a 70% ethanol solution containing 10% acetic acid for 40 s. The sections were then dehydrated in ethanol solutions (96% and twice in 100%) and incubated in two mixtures of alcohol and xylene (first mixture: two parts ethanol and one part xylene; second mixture: one part alcohol and two parts xylene), followed by two rinses in xylene. The sections were mounted using DePeX (Serva, Heidelberg, Germany).

### 2.4. Quantitative Analysis of Nissl Staining

Paraffin sections stained with cresyl violet were imaged using a Zeiss Axiovert 135 microscope (Zeiss, Jena, Germany). The frontal cortex was defined as region of interest according to stereotaxic coordinates [43] using the NIS X-Y plotting system (NIS-Elements BR 4.30.00, Laboratory Imaging s.r.o., Nikon Corporation, Prague, Czech Republic). Neurons were classified as neurodegenerative if they exhibited uniformly dark, shrunken cell bodies. The number of Nissl-stained cells exhibiting degenerative characteristics was counted manually using Fiji-ImageJ (v. 1.53t, NIH, Bethesda, MD, USA).

### 2.5. Quantitative Analysis of Immunohistochemical Staining

TH- and VMAT-2-immunoreactive neurons in SNpc were imaged using a Zeiss Axiovert 135 microscope (Zeiss, Jena, Germany). The anatomical area of SNpc was delineated as the region of interest using the NIS software X-Y plotting system. This system also measured the area of the region of interest in mm^2^. The number of TH- and VMAT-2 immunoreactive neurons was counted manually under 100× magnification. Only those neuronal bodies that demonstrated immunoreactivity were counted, and simultaneously the nuclei and proximal segments of one or two dendrites were distinctly discernible. The data concerning TH- and VMAT-2-immunopositive dopaminergic cells were presented as the number of cells within the region of interest for each brain section analyzed.

Immunohistochemical staining in frontal cortex and striatum was quantified as relative optical intensity (ROI) of labeling for all individual antibodies. Regions of interest were delineated, and the mean ROI was determined using the Fiji plug-in of the ImageJ software. Mathematically, the mean ROI was calculated as the sum of grey values of all pixels within the region of interest, divided by the number of pixels. Subsequently, thresholding was performed: the specified ROI value at which all immunolabeled structural tissue elements within the region of interest were selected over background labeling by ImageJ was identified and referred to as Threshold. This procedure was performed on a single occasion for the selected reference image. This threshold value was then utilized to analyze all other images. Given that each region of interest may not stain homogeneously, we also assessed the %Area as the ratio of the number of pixels in each region of interest with an ROI value below the threshold to the total number of pixels in the scrutinized region of interest.

All relevant experimental and imaging conditions were kept consistent to ensure comparability of all obtained measurements. These included section thickness; antibody incubation time and conditions, using antibodies from the same batch and processed in a single reaction; antibody concentrations; DAB development time; microscope illumination; camera settings, including exposure and white balance; and the method of slide preparation. The conditions were standardized precisely to reduce technical variability between samples.

Researchers were not blinded during the phase of histological analyses. The lack of blinding during histological analyses is a limitation of this study, which may have introduced observer bias and should be considered when interpreting the histological findings.

### 2.6. Statistical Analysis

The results of both accelerated and constant rotation velocity RotaRod tests were analyzed using linear mixed-effects models (LMMs) fitted using restricted maximum likelihood (REML) via GraphPad Prism. The assumption of sphericity was evaluated using Greenhouse–Geisser’s epsilon. Post hoc pairwise comparisons were conducted using Tukey’s HSD adjustment for multiple comparisons. For the inverted horizontal grid, comparisons between mean values were analyzed using one-way ANOVA with genotype as the between-subject factor. The quantitative data obtained from the immunohistochemical staining analyses (number of immunopositive neurons and staining intensity) were analyzed using one-way ANOVA. The significance level was set at *p* < 0.05. Statistical analysis was performed using GraphPad Prism software (version 9.5.1 (733)). Data are presented as mean with standard deviation (SD) and individual results are shown as scatter (bar charts). Only significant terms are given for clarity.

## 3. Results

### 3.1. Motor Phenotypes in L66 Mice

#### 3.1.1. Motor Learning and Coordination in the Accelerated RotaRod Test

To assess motor learning (incremental performance over trials/days) and motor coordination (performance on day 1), NMRI wild-type mice, L66-Het and L66-Homo transgenic tau mice underwent training on the accelerating RotaRod test over four consecutive days. Each day consisted of four trials, totaling 16 trials per mouse.

**Figure 2:**
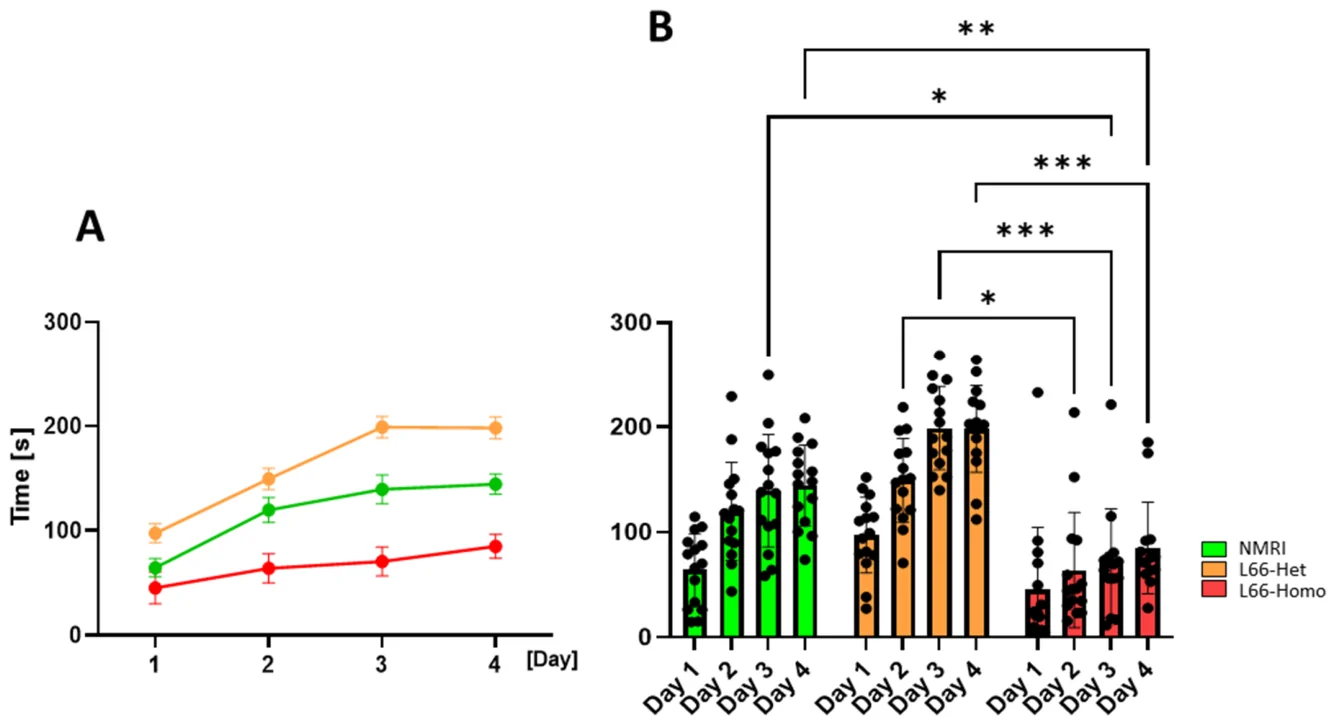
**Motor learning and coordination on the accelerated RotaRod.** Cohorts: NMRI, n = 15; L66-Het, n = 15; L66-Homo, n = 15. (**A**) Latency to fall in L66-Het and L66-Homo mice during training on the accelerated RotaRod for four consecutive days. L66-Homo mice demonstrated impaired performance across all days; L66-Het mice performed better than wild-type and L66-Homo. Yet all genotypes improved their performance over the training days (main effect of day F(1.388,19.43) = 72.39, *p* < 0.0001). (**B**) The effect of genotype on locomotor performance in the accelerated RotaRod test compared on consecutive days of the test (main effect of genotype F(1.818,25.45) = 23.87, *p* < 0.0001). The performance of L66-Homo mice on Day 1 was similar to that of NMRI and L66-Het mice. Analyses within each genotype revealed significantly improved performance on day 4 compared to day 1 in both the wild type and the L66-Het mice (*p*’s < 0.0001). In contrast, L66-Homo showed only a slight improvement that did not reach statistical significance. This slight improvement in L66-Homo may stem from impaired motor learning ability or limitations in motor performance that blunt the effect of motor learning. Data are presented as mean ± SD, with individual data points visualized as scatter. Statistical post hoc comparisons between genotypes and across days were performed using Tukey’s HSD adjustment for multiple comparisons (* *p* < 0.05, ** *p* < 0.01, *** *p* < 0.001).

Homozygous L66 mice exhibited pronounced deficits in task performance throughout the testing period, characterized by significantly shorter latencies to fall when compared to both NMRI and L66-Het cohorts (Figure 2A). This result suggests a marked impairment in motor learning and coordination in 9-month-old L66-Homo mice. L66-Het mice, in contrast, performed significantly better than L66-Homo mice and exhibited numerically higher latencies to fall than NMRI controls; however, the difference between L66-Het and NMRI mice did not reach statistical significance.

Values of Greenhouse–Geisser epsilon for day and day x genotype indicated a violation of sphericity (*ϵ* = 0.4626 and *ϵ* = 0.5794 respectively); accordingly, REML modeling was employed. There was a statistically significant main effect of day F(1.388,19.43) = 72.39, *p* < 0.0001 and a statistically significant main effect of genotype F(1.818,25.45) = 23.87, *p* < 0.0001. The day × genotype interaction was also statistically significant, F(3.476,47.51) = 6.95, *p* = 0.0003, confirming reduced learning in the L66-Homo group (see Figure 2B). Post hoc pairwise comparisons using Tukey’s HSD adjustment for multiple comparisons confirmed significant differences, primarily between the L66-Homo group and the two other cohorts, already strongly apparent on day 2 (Figure 2B).

#### 3.1.2. Locomotor Performance in the Constant Speed RotaRod Test

**Figure 3:**
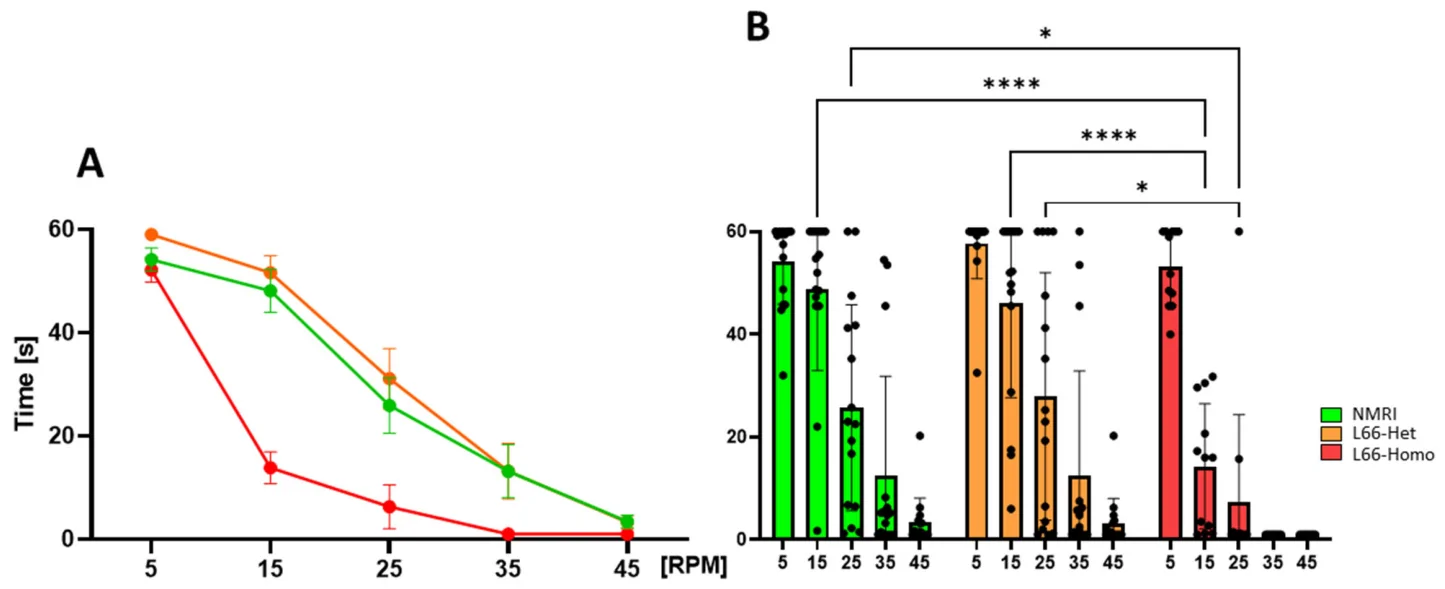
**Latency to fall in the constant velocity RotaRod protocol.** Cohorts: NMRI, n = 15; L66-Het, n = 15; L66-Homo, n = 15. (**A**) All genotypes were tested at 5 progressively increasing rotation speeds. Although all genotypes readily performed at 5 rpm, they were unable to sustain the maximum speed of 45 rpm. NMRI and L66-Het clearly outperformed L66-Homo mice in the middle range of rotation speeds (15–35 rpm). For overall statistics, see Results. (**B**) The same data are presented as phenotype-specific performances with individual data scatter and comparison between individual rotation velocities (see asterisks based on post hoc Tukey’s tests (* *p* < 0.05 and **** *p* < 0.0001). No significant difference was revealed between L66-Het and NMRI wild-type. Values are presented as the mean ± SD.

In a second experiment we assessed endurance on the rod rotating at constant speeds. In each trial, the rod was maintained at constant speed for 1 min and after a short ITI, the next trial proceeded with a higher rotation velocity. Data are summarized in Figure 3.

While all genotypes readily completed the first trial at 5 rpm with near-maximum times, L66-Homo mice already found 15 rpm extremely challenging and 25 rpm or faster was impossible to sustain. Although wild-type and L66-Het mice also struggled at 45 rpm, they were still able to perform at 15–25 rpm and only fell off at 35–45 rpm in less than 15 s. These results provide compelling support for the notion that L66-Het mice are not generally impaired, but L66-Homo mice aged 9 months are severely impaired at higher speeds.

Values of Greenhouse–Geisser epsilon for rotation velocity and rotation velocity x genotype again indicated a violation of sphericity (*ϵ* = 0.6403 and *ϵ* = 0.4611 respectively); consequently, REML modeling was employed. There was a statistically significant main effect of rotation velocity F(2.561,38.42) = 117.2, *p* < 0.0001 and a main effect of genotype F(1.891,28.37) = 12.78, *p* = 0.0001. The rotation velocity × genotype interaction was also statistically significant, F(3.689,46.11) = 4.465, *p* = 0.0048 (Figure 3A). Post hoc pairwise comparisons using Tukey’s HSD adjustment for multiple comparisons confirmed that L66-Homo mice performed significantly worse than L66-Het and NMRI mice at the mid-range of rotation velocities (15–25 rpm) (*p* < 0.001). This finding may be explained by limitations in motor performance: at the lowest rotation velocity, L66-Homo mice were still able to stay on the RotaRod; however, an increase in rotation velocity by 10 rpm caused a drastic decline in latency to fall, whereas the decline was only modest in NMRI and L66-Het mice. At the maximum rotation velocity, the latter animals were also unable to stay on the RotaRod, seemingly approaching the performance of the L66-Het. Taken together, the results obtained with the accelerated and constant rotation velocity RotaRod test can be explained by limitations in motor performance in L66-Homo mice. Figure 3B presents the same data set as a bar chart with group scatter and statistical post hoc comparisons. The reduced motor function exhibited by L66-Homo mice does not appear to be attributable to their reduced body weight (see Figure S1). Body weight was highest in NMRI mice, average in L66 Het mice, and lowest in L66 Homo mice, whilst motor performance was clearly superior in NMRI and L66 Het mice in comparison to L66 Homo mice.

#### 3.1.3. Traction Test

In rodents, and particularly mice, the basal ganglia play a crucial role in modulating forepaw motor function, while exerting a comparatively minor influence on hind limb movements [44]. Accordingly, behavioral paradigms that emphasize forepaw-dependent manipulations are well-suited for detecting motor impairments associated with dysfunction of the nigrostriatal dopaminergic pathway. The Traction test, also known as the Inverted Horizontal Grid test, is widely employed to probe this function and was used in this study. Automated quantification of multiple metrics was performed using the validated TracMouse script [29].

**Figure 4:**
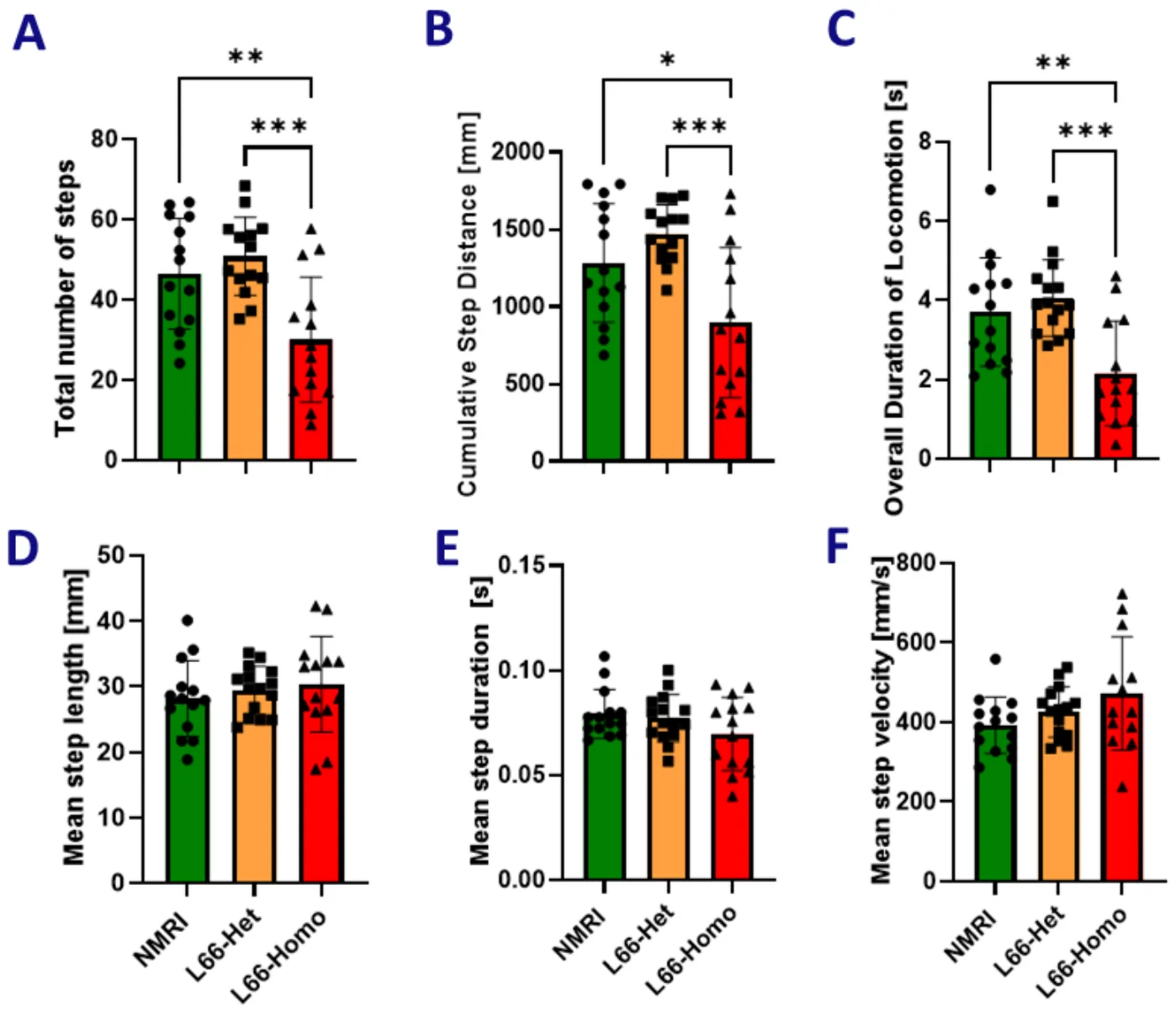
**Forepaw movement characteristics in the Inverted Horizontal Grid.** The mean values (±SD) of step-related indices are given for NMRI wild-type mice (NMRI, n = 14), L66-Het (n = 14 in (**A**,**B**,**F**) and n = 15 in (**C**–**E**)) and L66-Homo (n = 14) mice: (**A**) Total number of Steps; (**B**) Cumulative Step Distance [mm]; (**C**) Overall Duration of Locomotion [s]; (**D**) Mean Step Length [mm]; (**E**) Mean Step Duration [s]; (**F**) Mean Step Velocity [mm/s]. See Table S1 for detailed definitions. Statistical significance between the groups was calculated using Tukey post hoc test following ANOVA (see Results; * *p* < 0.05, ** *p* < 0.01 and *** *p* < 0.001).

During the Inverted Horizontal Grid test, exploratory movements with the front paws occurred more frequently, while hind paws remained stationary for longer and showed less displacement. Consequently, the data from the front and hind paws were presented separately. Quantitative analysis of forepaw movement patterns in the Inverted Horizontal Grid revealed pronounced motor deficits in L66-Homo mice relative to both NMRI wild-type and L66-Het counterparts (Figure 4). Statistical comparison of the three genotypes returned significant differences for L66-Homo mice for reduced Total Number of Steps (F(2,39) = 9.544, *p* < 0.001), cumulative distance traversed by forepaws (F(2,39) = 8.586, *p* < 0.001), and the Overall Duration of Locomotion (F(2,40) = 9.743, *p* < 0.001). The source of these effects was identified as L66-Homo mice, which significantly differed from both wild-type and L66-Het cohorts by expressing lower values for all parameters (see asterisks in Figure 4A–C). No other post hoc comparison was significant.

However, when the characteristics of individual steps were analyzed (Figure 4D–F), no differences were observed between genotypes. Therefore, it can be concluded that the impairment is not limited to the movement of individual forepaws but rather affects the coordination of locomotion in its entirety. This impairment was observed only in L66-Homo mice. The data obtained in this study are consistent with those from previous experiments on the RotaRod. These experiments also clearly demonstrate that abnormally slow movements are difficult to differentiate between L66 and wild-type mice. However, L66-Homo mice are unable to cope if challenged by increased rotational speed or hanging movements. It is important to note that these movements are widely executed during their normal life in home cages.

### 3.2. Tyrosine Hydroxylase (TH) and Vesicular Monoamine Transporter (VMAT-2) Are Reduced in L66-Homo in Substantia Nigra Pars Compacta, Striatum and Frontal Cortex

**Figure 5:**
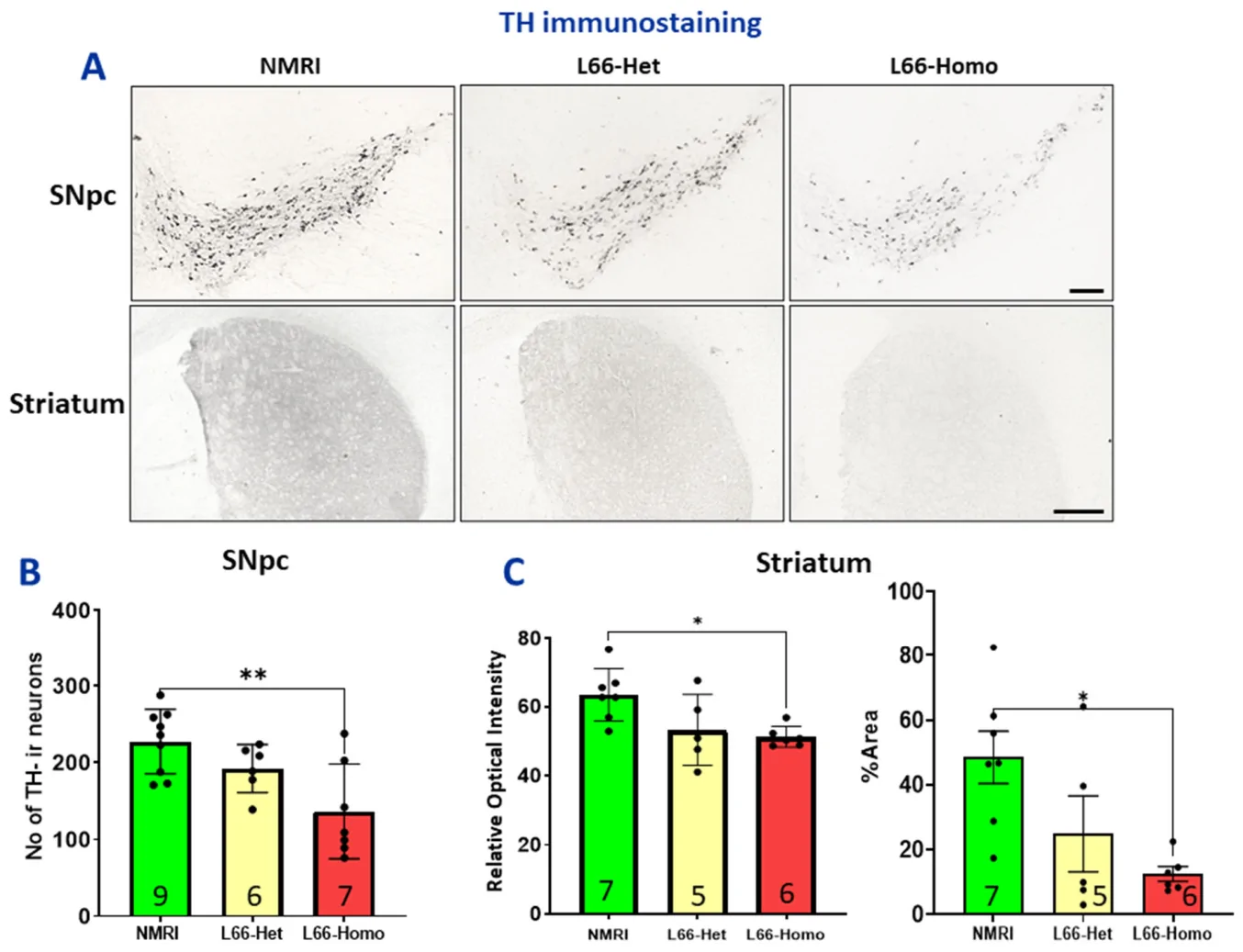
**Tyrosine hydroxylase (TH) staining in brains of wild-type NMRI (n = 7–9 depending on section quality), L66-Het (n = 5–6) and L66-Homo mice (n = 6–7).** (**A**) Representative microphotographs of anti-TH staining demonstrated high abundance of immunolabeling in SNpc neurons and in neuropil of the striatum in NMRI and L66-Het mice. Considerably less staining was observed in L66-Homo tissue. (**B**) The mean number of TH-immunopositive neurons in the SNpc was significantly reduced in L66-Homo mice relative to wild-type only (asterisks). (**C**) The intensity of anti-TH labeling in the striatum, as measured by mean ROI (**left**) and %Area (**right**), was reduced in both L66 cohorts. Significances were returned only for L66-Homo mice relative to wild-type NMRI. For data obtained from one-way ANOVA, see Results. Values are presented as the mean ± SD with scatter for individual data points. Statistical significance between the groups was calculated using the post hoc Tukey test (* *p* < 0.05, ** *p* < 0.01). Numbers inside the bars represent group sizes. Scale bars: 100 µm for the substantia nigra pars compacta and 500 μm for the striatum.

A marked attenuation in the intensity of TH immunostaining was observed exclusively in the L66-Homo group. Relative to wild-type NMRI mice, a small lowering was also observed in L66-Het mice, which did not reach significance. This may be a gene dosage effect, as the lowering in substantia nigra cell count and striatum relative optical intensity (ROI) and area affected were around half of the amount lost in L66-Homo (Figure 5A–C).

The primary focus of this study was the number of TH-positive neurons in the substantia nigra pars compacta (SNpc), for which the analysis yielded a main effect of genotype (F(2,19) = 7.423, *p* < 0.01) (Figure 5B). Similar results were observed for mean ROI and %Area in the striatum (Figure 5C) (F(2,15) = 5.106, *p* < 0.05 and F(2,15) = 5.768, *p* < 0.05). Sources of these variations were explored using Tukey’s post hoc test and are presented in Figure 5B,C (asterisks). They confirm that L66-Homo mice expressed much reduced dopamine markers relative to wild-type controls.

**Figure 6:**
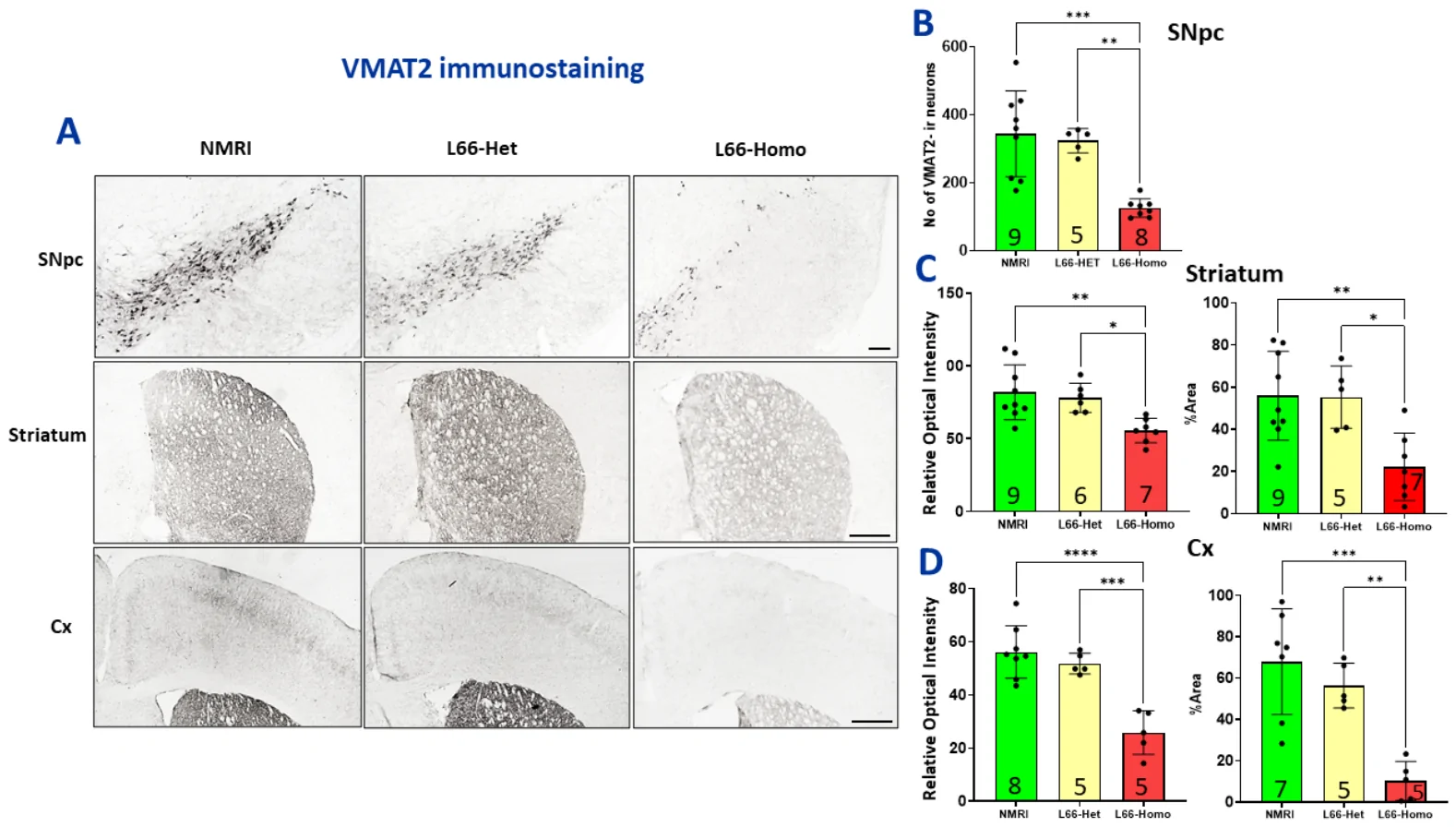
**Immunohistochemical staining for vesicular monoamine transporter 2 (VMAT-2) in the brains of wild-type NMRI (n = 7–9 depending on section quality), L66-Het (n = 5–6) and L66-Homo mice (n = 5–8).** (**A**) Representative micrographs of anti-VMAT-2 staining demonstrate an abundance of immunolabeling in SNpc neurons and in the neuropil of both striatum and frontal cortex in NMRI and L66-Het mice. Tissue staining is much less prominent in the L66-Homo group. The brain areas in question were then subjected to quantification, with the number of VMAT-2-immunopositive neurons in the SNpc being recorded in (**B**). The ROI and area covered were determined in the striatum (**C**) and the frontal cortex (Cx) (**D**). All proxies returned main effects of genotype (see Results), and post hoc Tukey’s test confirmed low expression in L66-Homo mice, compared with wild-type and L66-Het, as the only source of this effect (see asterisks). Values are presented as the mean ± SD with scatter of individual data points. * *p* < 0.05, ** *p* < 0.01, *** *p* < 0.001, **** *p* < 0.0001. Numbers inside the bars represent group sizes. Scale bars: 100 µm for the SNpc and 500 μm for the striatum and frontal cortex.

A confirmatory analysis was conducted to measure monoaminergic signaling in the same brain structures with an antibody for the vesicular monoamine transporter 2 (VMAT-2). Representative micrographs are presented in Figure 6A and show dense VMAT-2 immunoreactivity in SNpc neurons and the neuropil of the striatum and cortex in tissue from wild-type and L66-Het animals. By contrast, L66-Homo mice exhibited a marked reduction in labelling intensity, suggesting that higher expression of human tau caused a strong depletion of presynaptic monoamine transport capacity.

The quantification of VMAT-2-positive neurons and fibers confirmed this impression. In SNpc there was a reliable difference between genotypes (F(2,19) = 15.81, *p* < 0.0001) with only the L66-Homo group being affected (see asterisks in Figure 6B). The same pattern was reproduced for the striatum (Figure 6C—mean ROI: F(2,19) = 7.545, *p* < 0.01, %Area: F(2,18) = 7.883, *p* < 0.01) and the frontal cortex (Figure 6D—mean ROI: F(2,15) = 22.30, *p* < 0.0001, %Area: F(2,14) = 15.10, *p* < 0.001). The respective post hoc tests (Tukey’s) all confirmed no differences between wild-type NMRI and L66-Het, but both groups differed significantly from the strongly reduced L66-Homo, thereby reinforcing the notion of widespread synaptic alterations in the dopaminergic system in homozygous L66 mice.

Collectively, these results underline a substantial compromise in monoaminergic integrity associated with the L66 homozygous genotype, extending beyond the nigrostriatal system and encompassing cortical circuits as well. Whether these alterations are due to the expression of transgenic human tau was investigated next.

### 3.3. Enhanced Tau Immunoreactivity in Striatum and Frontal Cortex of Homozygous L66 Transgenic Mice

After confirmation that dopamine pathways were strongly reduced in L66-Homo mice, it was important to determine the levels of human tau expression as a potential explanation for this decline. This was investigated with three tau-specific antibodies, 7/51, S-1D12, and AT8, for the SNpc, striatum (ST) and frontal cortex (Cx).

#### 3.3.1. Tau Immunostaining Using Antibody 7/51

**Figure 7:**
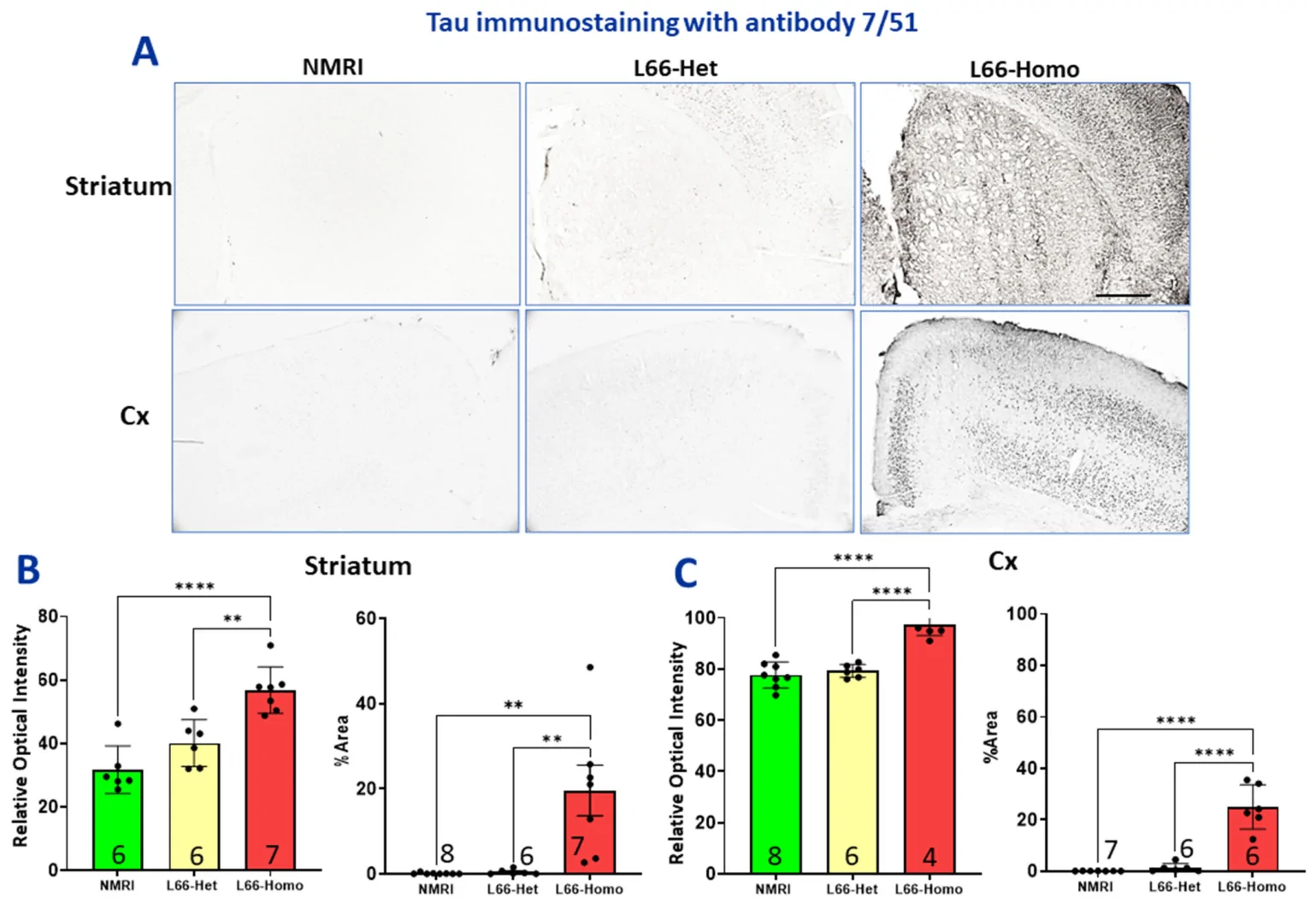
**Tau immunolabelling using antibody 7/51 in striatum and frontal cortex (Cx). Wild-type NMRI (n = 6–8 depending on section quality), L66-Het (n = 6), L66-Homo (n = 4–7).** (**A**) Representative micrographs of tau labelling demonstrate an abundance of immunolabeling in the striatal neuropil and frontal cortex somata of L66-Homo mice. This phenomenon was particularly evident in pyramidal neurons located in layers three, five and six of the cerebral cortices. The staining intensity was found to be minimal in the L66-Het group, with no labeling observed in NMRI mice. (**B**) The intensity of tau labeling in the striatum, quantified as mean ROI (**left**) and %Area (**right**). Labelling intensity was highest in L66-Homo mice. (**C**) Tau staining in the frontal cortex, measured as mean ROI (**left**) and %Area (**right**). Again, labelling intensity was highest in L66-Homo mice. Note that mAb 7/51 recognizes both murine and human tau proteins. Values are presented as the mean ± SD with scatter for individual data points. Statistical significance between the groups was based on ANOVA (see main Results) and post hoc Tukey test (** *p* < 0.01, **** *p* < 0.0001). Numbers inside the bars represent group sizes. Scale bar: 500 μm.

Microscopic examination revealed robust tau immunoreactivity within the striatal neuropil and frontal cortical neurons of L66-Homo mice. Immunolabeling was especially prominent in pyramidal neurons located in cortical layers III and V–VI. In contrast, L66-Het mice exhibited only weak staining, and tau signals were not detectable in NMRI control mice (Figure 7A). We quantified the tau pathology in the two regions by measuring the mean relative optical intensity (ROI) and percentage area covered (%Area). Both metrics demonstrated significantly elevated levels of tau in L66-Homo mice compared to L66-Het and NMRI groups (Striatum: ROI: F(2,16) = 19.59, *p* < 0.0001, %Area: F(2,18) = 10.66, *p* < 0.001; frontal cortex: ROI: F(2,18) = 47.53, *p* < 0.0001, %Area: F(2,16) = 50.32, *p* < 0.0001). Post hoc comparisons confirmed that mean ROI levels were significantly higher in L66-Homo mice, but no differences were revealed between NMRI controls and L66-Het mice. Similar group differences were observed in %Area (see asterisks in Figure 7B,C).

#### 3.3.2. Tau Immunostaining Using the S-1D12 Antibody

Using a second antibody, we sought to confirm the results with mAb 7/51 described above. Antibody S-1D12 (TauRx Therapeutics Ltd., Aberdeen, UK) recognizes the 337–355 epitope of the tau molecule of both human and murine origin, and strongly labels neuronal processes predominantly in dendrites and axons throughout the brain [45].

**Figure 8:**
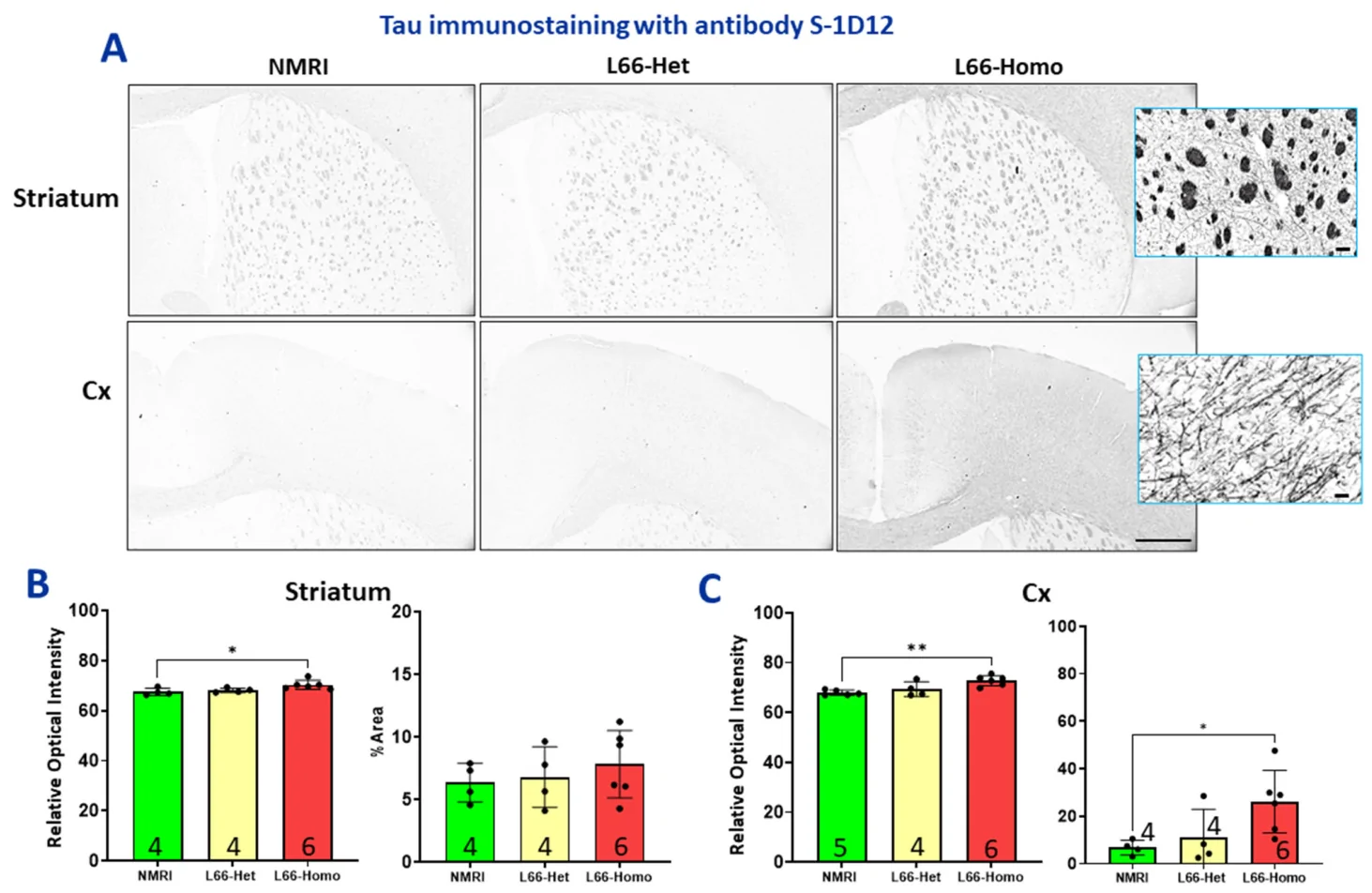
**Tau immunolabelling using antibody S-1D12 in striatum and frontal cortex (Cx). Wild-type NMRI (n = 4–5 depending on section quality), L66-Het (n = 4), L66-Homo (n = 6).** (**A**) Representative micrographs of S-1D12-positive staining demonstrate that there is an abundance of immunolabelling in the neuronal processes of both striatum and frontal cortex, which is seemingly heightened in L66-Homo mice. Considerable labelling, however, was also revealed in wild-type mice, suggesting that this antibody effectively labelled both human and murine tau proteins. The intensity of tau labeling in the striatum (**B**) and frontal cortex (**C**) confirmed this impression. While mean ROI values significantly differed in L66-Homo mice compared with wild-type, this was only the case for %Area in frontal cortex, but not striatum (see asterisks; for overall ANOVA, see main Results). Values are presented as mean ± SD with scatter for individual data points. Statistical significance between the groups was calculated using one-way ANOVA followed by post hoc Tukey’s test (* *p* < 0.05, ** *p* < 0.01). Numbers inside the bars represent group sizes. Scale bars: The dimensions for the main microphotographs are 500 µm, while the inserts for the striatum and frontal cortex are 100 µm and 50 µm, respectively.

Microscopic analysis of S-1D12-labelled brain sections revealed an accumulation of tau-immunoreactivity within neuronal processes in both the striatum and frontal cortex of L66-Homo mice. In contrast, less immunolabelling was detected in tissues from wild-type NMRI and L66-Het animals, indicating substantially lower tau burden in these genotypes (Figure 8A). Consequently, quantitative analysis of the striatum (Figure 8B) yielded an overall effect only for mean ROI (F(2,11) = 4.773, *p* < 0.05), but not for %Area (F < 1). This contrasted with the frontal cortex, for which both mean ROI and %Area were significant (F(2,12) = 8.133, *p* < 0.01 and F(2,11) = 4.394, *p* < 0.05, respectively) (Figure 8C). Post hoc Tukey’s tests were used to confirm the L66-Homo group as a source of variation, with heightened levels being observed (see asterisks in Figure 8B,C).

#### 3.3.3. Immunostaining of Phosphorylated Tau Using the Antibody AT8

**Figure 9:**
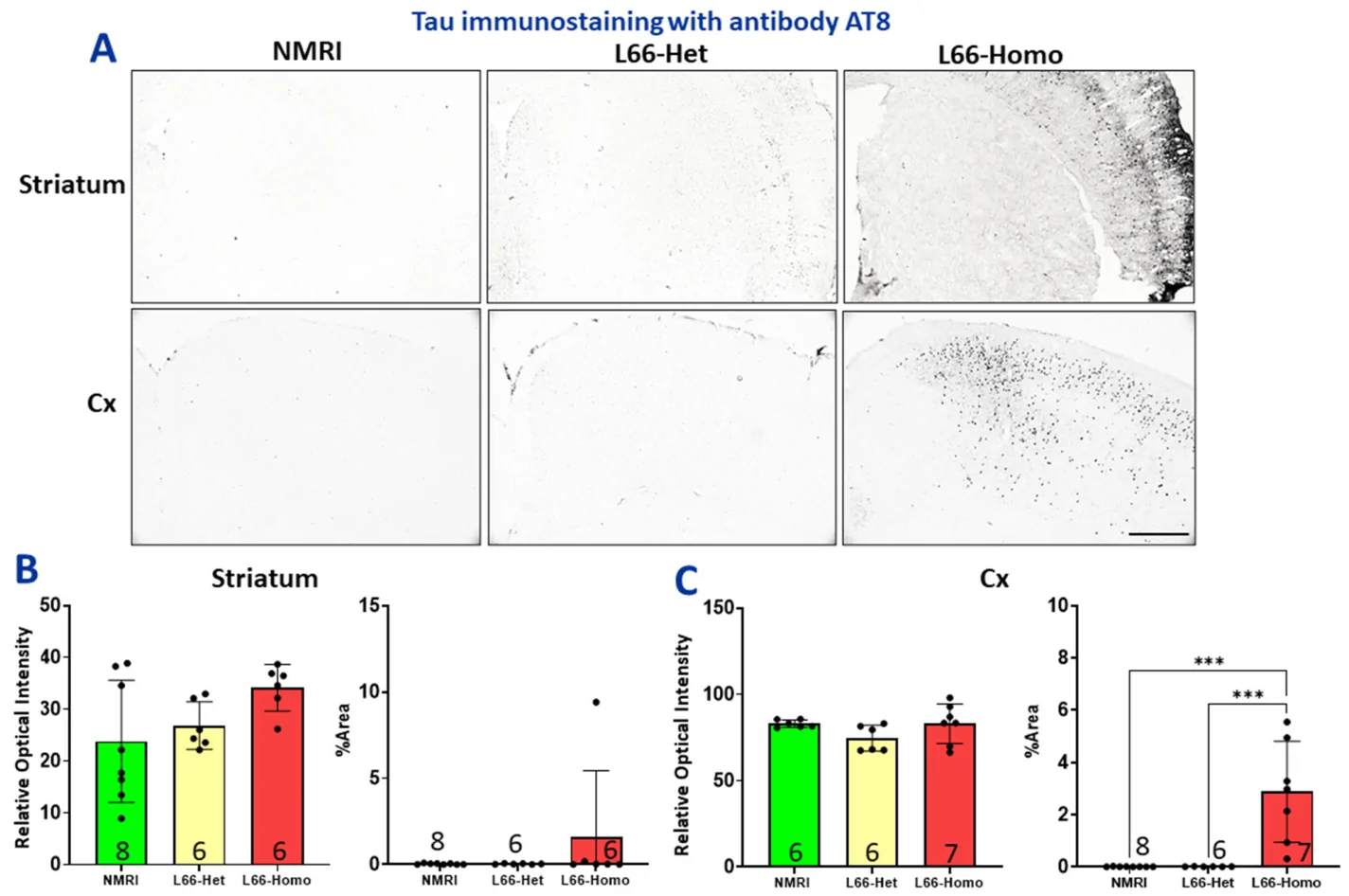
**AT8 labelling of phosphorylated tau in striatum and frontal cortex (Cx). Wild-type NMRI (n = 6–8 depending on section quality), L66-Het (n = 6), L66-Homo (n = 6–7).** (**A**) Representative micrographs of AT8-immunolabelled brain sections of neuronal processes in striatum and neuronal cell bodies in frontal cortex in L66-Homo mice. Lower levels of anti-tau staining were found in L66-Het and NMRI wild-type groups. (**B**) Quantification of AT8 labelling in the striatum measured by mean ROI and %Area. While L66-Homo mice showed higher staining intensities, this was not significantly different from either L66-Het or wild-type. (**C**) Staining in frontal cortex was also higher in L66-Homo mice but only differed significantly for %Area (for ANOVA, see Results). Values are presented as mean ± SD with scatter for individual data points. Statistical significance between the groups was calculated using the post hoc Tukey test (*** *p* < 0.001). Numbers inside the bars represent group sizes. Scale bar: 500 µm.

The literature consistently shows that abnormally high levels of phosphorylated tau are a core pathological feature of tauopathies. These findings are supported by a strong consensus from molecular, neuropathological, and clinical research [46,47,48]. Consequently, we labeled abnormal phosphorylated tau with AT8 in our three genotypes. Microscopic analysis of stained brain sections showed pronounced tau positivity in the neuronal processes of the striatum and somata of the frontal cortex in L66-Homo animals, whereas staining was notably weaker in L66-Het and NMRI wild-type controls (Figure 9A).

Quantification for the striatum (Figure 9B) showed increased levels of AT8 labelling in L66-Homo mice, but the group comparisons for mean ROI or %Area did not reach significance (F’s < 3, *p’s* = NS). Similar results were obtained for mean ROI in frontal cortex (Figure 9C) but %Area was significantly different between groups (F(2,18) = 15.36, *p* < 0.001) due to hugely enhanced AT8 labelling in L66-Homo mice (see asterisks).

#### 3.3.4. Tau Immunostaining in the Midbrain

**Figure 10:**
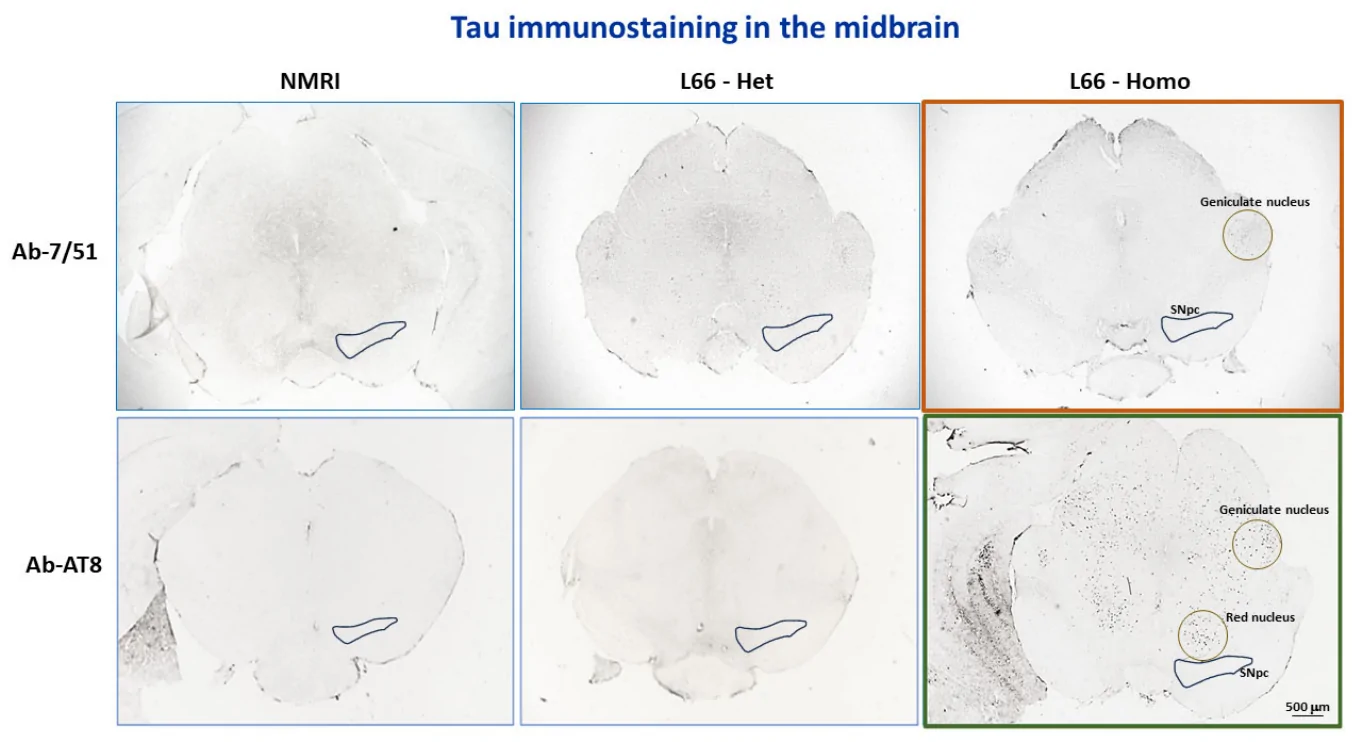
**Representative microphotographs of midbrain sections labelled with the antibodies 7/51 and AT8 in NMRI, L66-Het, and L66-Homo mice.** There was a lack of 7/51 and AT8 tau staining in the substantia nigra (SNpc in blue frames) across all the analyzed groups. In contrast, immunolabeling of tau protein was observed in other midbrain structures in L66-Homo mice, including the red nucleus (labelled with AT8, green frame) and the geniculate nucleus (labelled with both AT8 (green) and 7/51 (orange frame)). Scale bar: 500 µm.

Furthermore, we examined tau immunolabelling in midbrain structures, focusing on the substantia nigra because of its efferent connections with the striatum. No tau-positive labelling with either the 7/51 or AT8 antibodies was observed in the substantia nigra in any experimental group. In contrast, robust tau immunoreactivity was detected in the lateral geniculate nucleus and the red nucleus (Figure 10). Specifically, AT8 immunoreactivity was restricted to the red nucleus, whereas both AT8 and 7/51 antibodies labelled tau accumulation in the geniculate nucleus (Figure 10, right panel). These data were not quantified due to the lack of staining in the substantia nigra as our primary outcome.

### 3.4. Does Tau Protein Aggregation Initiate Neuronal Death?

We next evaluated whether overexpression of tau protein induces overt neuronal loss. The preceding data suggest that the aggregation of tau protein led to neuronal degeneration, with subsequent emergence of movement anomalies. This was explored by estimating the number of neurons in the motor cortex and striatum using immunostaining for NeuN to label neuronal nuclei, and cresyl violet (Nissl) staining to visualize neuronal morphology in regions implicated in the observed motor phenotype.

#### NeuN and Cresyl Violet Staining in Motor Cortex and Striatum

**Figure 11:**
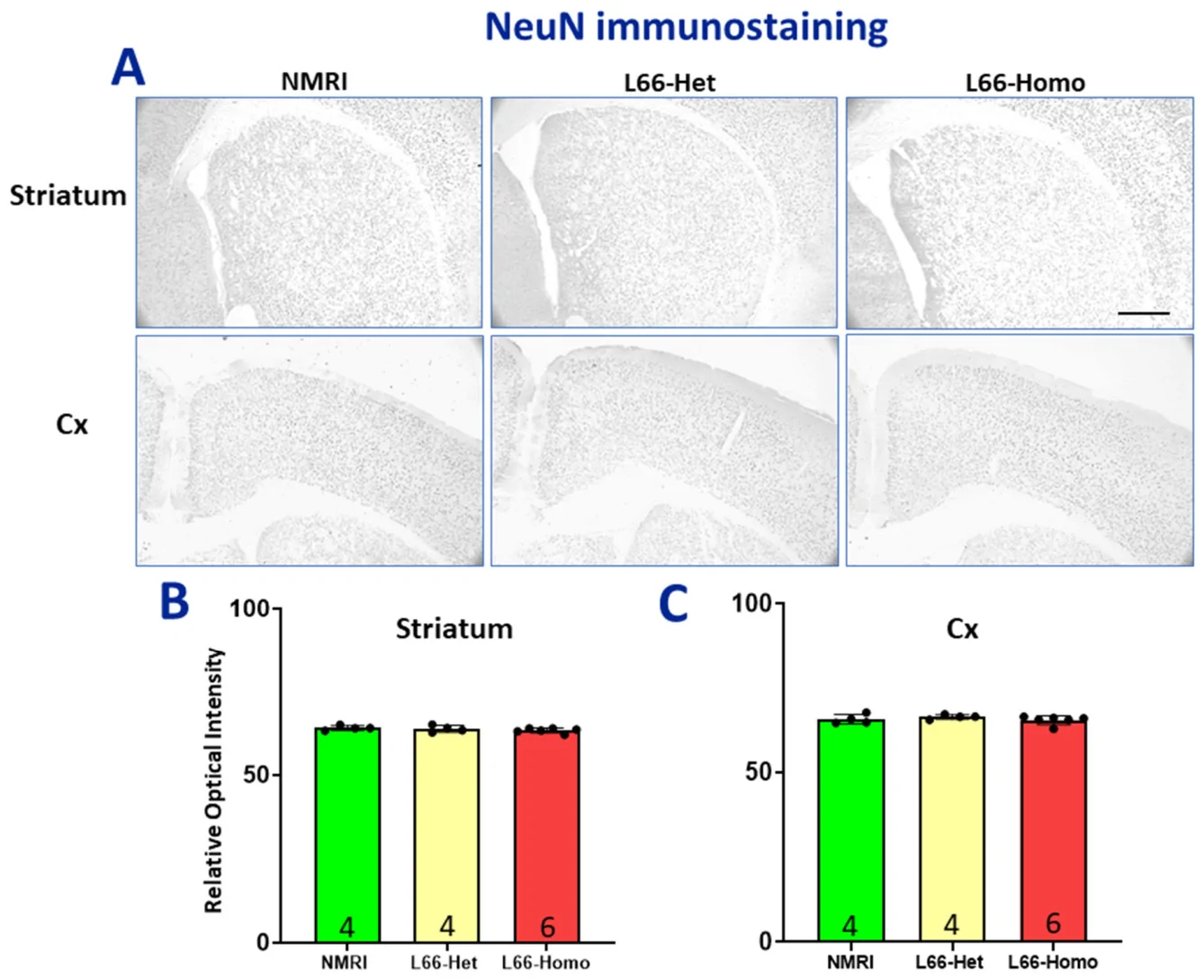
**Immunolabelling of neurons using antibody NeuN in the frontal cortex and the striatum. Wild-type NMRI (n = 4), L66-Het (n = 4), L66-Homo (n = 6).** (**A**) Representative NeuN-stained micrographs demonstrate that the intensity of immunolabeling in striatum and motor cortex was comparable in L66-Homo, L66-Het, and wild-type NMRI mice. The intensity of NeuN labelling in the striatum (**B**) and motor cortex (**C**) was similar when quantified as mean ROI and did not differ significantly between groups (F’s < 1). Values are presented as mean ± SD with scatter for individual data points. Numbers inside the bars represent group sizes. Scale bar: 500 µm.

NeuN-immunostaining showed robust nuclear labeling of neurons in both the motor cortex (primary—M1, and secondary—M2) and striatum across all three groups. Visual inspection of the micrographs indicated that the intensity and distribution of NeuN immunoreactivity were comparable between genotypes (Figure 11A) and quantification confirmed the absence of any differences (Figure 11B,C).

**Figure 12:**
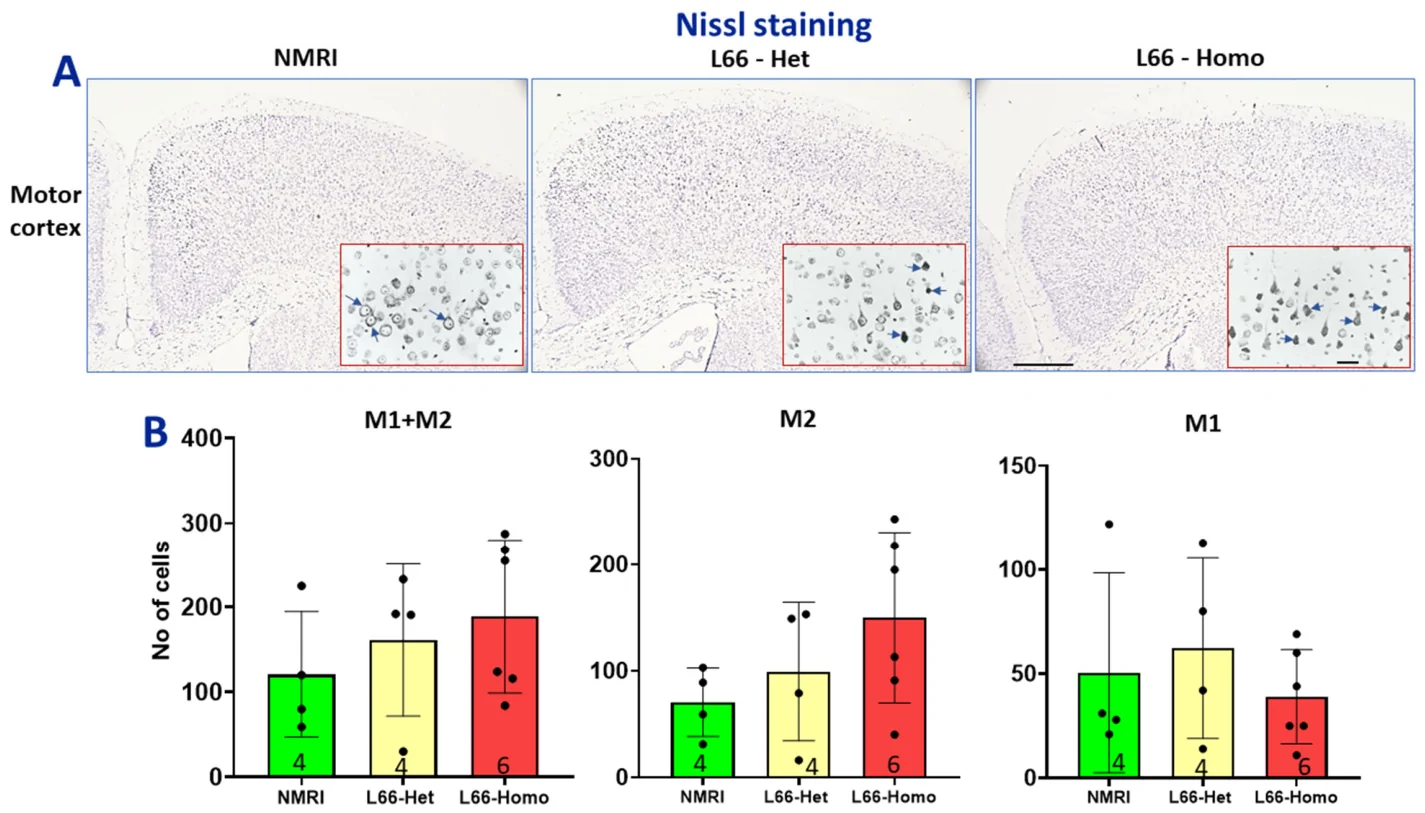
**Cresyl violet (Nissl) staining in primary (M1) and secondary (M2) motor cortex**. **Wild-type NMRI (n = 4), L66-Het (n = 4), L66-Homo (n = 6)**. (**A**) Representative micrographs of Nissl-stained sections of the motor cortex showing nerve cells exhibiting clear degenerative characteristics, such as shrunken neuron bodies and chromatin condensation. These were visible in all three experimental groups (see inserts, blue arrows); however, in controls, the number of abnormal neurons was comparatively low. (**B**) Quantification as mean number of neurons exhibiting degenerative characteristics was elevated in both L66-Het and L66-Homo mice in comparison to the NMRI control group. However, differences were not statistically significant. Values are presented as mean ± SD with scatter for individual data points. Numbers inside the bars represent group sizes. Scale bar: 500 µm.

Similar results were obtained for cresyl violet-stained cortical and striatal sections. Representative micrographs (Figure 12A) illustrate the typical appearance of Nissl-positive neurons across groups and highlight cells exhibiting overt degenerative features. These neurons exhibited shrunken somata, increased cytoplasmic basophilia and chromatin condensation, which are all consistent with neuronal injury. These abnormal neurons were visible in sections from all three experimental groups, although their frequency and distribution appeared to vary between animals and cortical subregions. Normal-appearing pyramidal and non-pyramidal neurons were also present in all groups, providing internal contrasts for the observed degenerative profiles. Quantitative counts of neurons exhibiting loss of cell markers were performed across the entire motor cortex, as well as separately for M1 and M2 areas. Despite regional differences in the number of cells, there was no effect of genotype (F’s < 1.9) (Figure 12B).

### 3.5. Loss of Cells, or Merely a Loss of Phenotype?—Tauopathy and Neurotransmitter Identity

The loss of dopaminergic projections from the substantia nigra to the striatum alters the function and plasticity of corticostriatal circuitry involved in motor control. Dopamine depletion modifies corticostriatal neurotransmission and the responsiveness of striatal medium spiny neurons to cortical inputs, contributing to alterations in the functional balance of basal ganglia circuits [49,50,51]. Given the previously observed reduction in dopaminergic markers in the striatum of L66-Homo mice, we therefore assessed glutaminase (GLS) expression in the striatum and motor cortex to determine whether tau pathology is also associated with alterations in glutamate metabolism within these regions. This approach was intended to provide further insight into potential neurochemical alterations affecting corticostriatal and cortico-subcortical motor circuitry.

**Figure 13:**
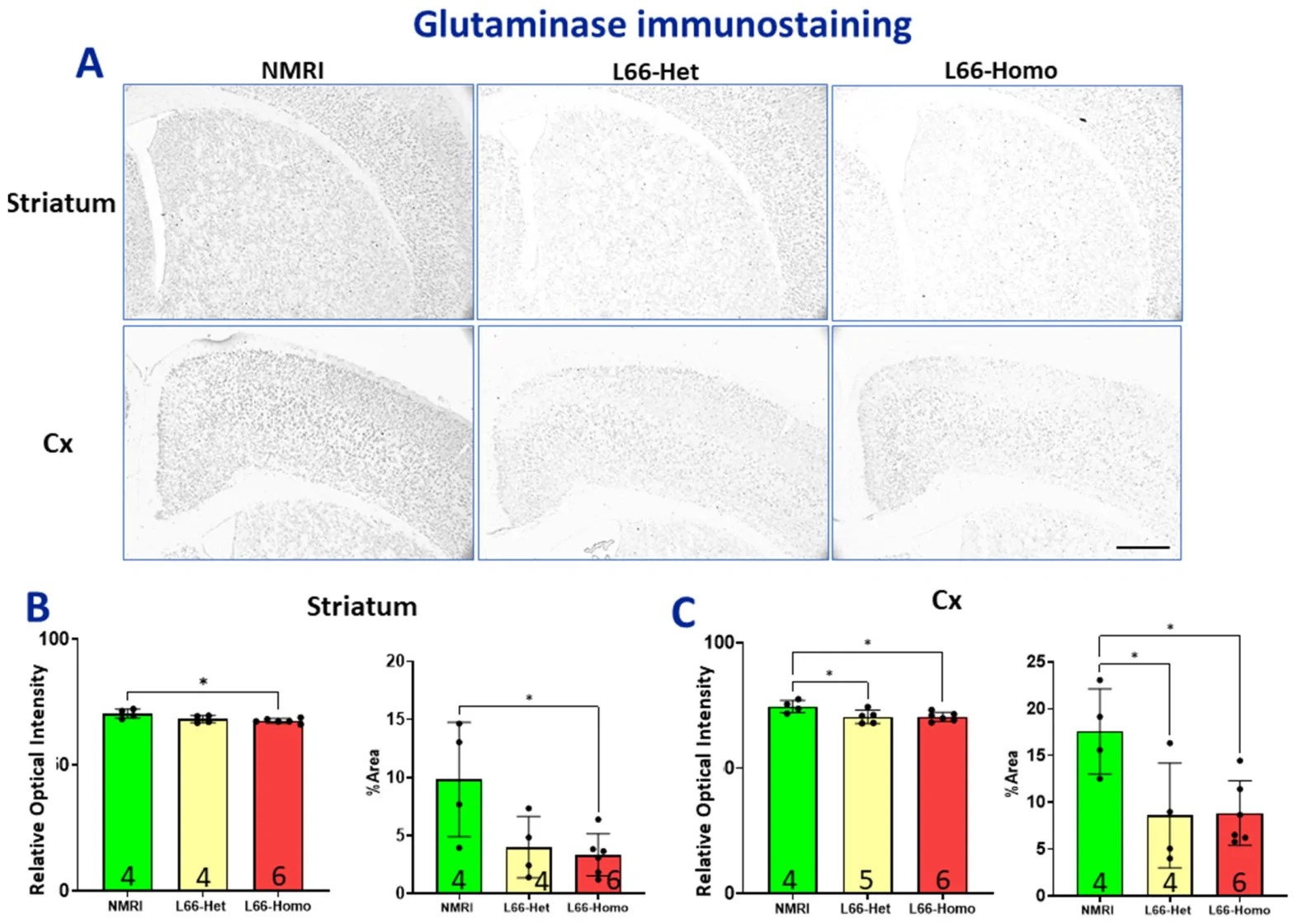
**Staining for glutaminase in the motor cortex and striatum. Wild-type NMRI (n = 4), L66-Het (n = 4–5 depending on section quality), L66-Homo (n = 6)**. (**A**) Representative micrographs of anti-glutaminase-stained sections demonstrate abundant immunolabeling in cortical neurons and striatal neuropil in NMRI mice. A marked reduction was evident in both L66-Het and L66-Homo groups. (**B**) The intensity of glutaminase labelling in the striatum, quantified as mean ROI and %Area, was lower in L66-Het and L66-Homo than in wild-type NMRI mice, but only reached significance in the L66-Homo cohort (see asterisks). (**C**) By contrast, this reduction in glutaminase also reached significance in L66-Het mice in motor cortex (see asterisks). Values are presented as the mean ± SD, with scatter for individual data points. Statistical significance between the groups was calculated using the post hoc Tukey test (* *p* < 0.05). Numbers inside the bars represent group sizes. Scale bar: 500 µm.

Glutaminase immunohistochemistry was performed on coronal sections containing the motor cortex and striatum from all three genotypes. Anti-glutaminase staining was robust in cortical neurons, and dense neuropil labelling in the striatum of NMRI mice was observed. In contrast, both L66-Het and L66-Homo groups presented with a marked reduction in staining intensity, with visibly fewer labeled somata in cortex and decreased neuropil signal in striatum (Figure 13A).

This impression was confirmed by quantification of the glutaminase signal. In the striatum, ROI and %Area measurements likewise showed significant differences between groups (ROI: F(2,11) = 5.602, *p* = 0.0210, %Area: F(2,11) = 5.575, *p* = 0.0213). In case of ROI pair-wise comparison revealed the significantly lower level anti-glutaminase staining in L66-Homo in comparison to NMRI (*p* = 0.0179). In the case of %Area, a significantly lower value was found in L66-Homo compared to NMRI (*p* = 0.0221). In the L66-Het group, the mean %Area (4.0%) was also markedly lower than in NMRI (9.8%), showing a strong trend toward reduction; however, this difference did not reach formal statistical significance (*p* = 0.0593). This lack of statistical significance is likely attributable to limited statistical power (54.9%) associated with the smaller sample size in the L66-Het group (n = 4) compared to the L66-Homo group (n = 6). Notably, no statistically significant difference was detected between L66-Het and L66-Homo (*p* = 0.9428), with both groups exhibiting closely aligned mean values (4.0% vs. 3.3%). While these data suggest that L66-Het animals display a phenotypic profile descriptively similar to L66-Homo, larger cohorts are required to definitively confirm this reduction (Figure 13B). Significant effects of genotype were shown for the motor cortex (ROI: F(2,12) = 4.770, *p* = 0.0299, %Area: F(2,11) = 5.710, *p* = 0.0199), and both L66-Het and L66-Homo animals presented with significantly lower values (Figure 13C, asterisks) relative to wild-type. These data indicate that tau expression in the motor cortex is more likely to induce loss of the glutamatergic phenotype and a consequent attenuation of excitatory transmission to striatal medium spiny neurons, rather than causing widespread frank neuronal cell loss.

## 4. Discussion

This is the first study on L66 mice to investigate the dopaminergic system in relation to motor performance, which is known to be impaired in patients suffering from heritable forms of FTDP-17. Our unexpected findings include that L66-homo mice were motorically impaired while L66-het mice were not, that dopaminergic markers are reduced in L66-homo, but much less in L66-het mice, and that tau expression can be readily detected in the motor system apart from the SNpc. These data are interesting in the context of drug development, which may not only target tau lowering, but also boost dopamine.

### 4.1. Comparison of Tau Species Recognized by 7/51, S-1D12 and AT8 Antibodies

Typically, P301S is a transgenic model that exhibits high levels of human tau expression under the Thy1 promoter [52]. It is characterized by very high sarcosyl-insoluble tau even at an early age, as well as the rapid accumulation of large amounts of tau filaments and massive tau aggregation [4,53]. These filaments are dense and homogeneous, resembling early paired helical filaments (PHFs) or protofilament-like structures with a higher degree of order [54,55]. In a significant proportion of P301S transgenic mouse lines (e.g., TAU58/2), there is an overexpression of human tau, which results in increased tau available for phosphorylation by kinases [53,56].

#### 4.1.1. Phosphorylation in the Proline-Rich Domain (PRD)

Phosphorylation in the proline-rich domain (PRD) and in the microtubule-binding domains at the C-terminus of tau predominates in the pattern of post-translational modifications. Hyperphosphorylation in these regions has been shown to be a key driver of normal, soluble tau transitioning into insoluble, aggregating species [53,57]. A study by Götz et al. [58] found that excessive accumulation of the insoluble sarkosyl fraction of tau led to a rapid increase in phospho-tau in the PRD. This chronological progression was confirmed in the present study by the use of the AT8 antibody, which recognizes tau phosphorylated at Ser202 and Thr205—an epitope located strictly within the PRD. In Alzheimer’s disease, AT8 labelling is observed at Braak stages I–II, representing the early stages of disease development [4]. Furthermore, in P301S cell lines, the extent of AT8 labelling correlates strongly with the appearance of tau in both fibers and the insoluble fraction [59]. These findings are highly consistent with the robust immunoreactivity to phosphorylated tau observed in our current model.

#### 4.1.2. Repeat Domain (RD) and Core Tau Region Structure

In order to evaluate tau pathology independently of its phosphorylation status, the focus was directed towards the microtubule-binding repeat domain (RD), which constitutes the structural core of tau filaments and paired helical filaments (PHFs). Classic biochemical studies by Wischik and colleagues [60,61], followed by recent high-resolution cryo-electron microscopy (cryo-EM) structural analyses [62,63], have established that the proteolytically stable core of aggregated tau resides precisely within this repeat region. In order to comprehensively capture these conformational changes, two distinct, phosphorylation-independent antibodies were utilized: 7/51 and S-1D12. It has been demonstrated that both antibodies recognize specific, overlapping epitopes at positions 343 to 355, which are located within the C-terminal repeat domain. The employment of two distinct antibodies against this region is predicated on two rationales: firstly, to ensure robust validation of the presence of the core tau assembly, and secondly, to account for potential steric hindrance or differential epitope accessibility that occurs when tau transitions from a monomeric state into complex, high-order fibrillar aggregates. The accumulation of these structural forms was successfully detected in the present studies. At 9 months of age, the L66 homozygous animals carrying the P301S mutation exhibited elevated levels of human tau, concomitant with the presence of abundant, structurally mature tau filaments in multiple brain regions [11]. These are derived from overexpression of human, but not a concomitant elevation of murine tau [64].

### 4.2. Relationship Between Motor Impairment and Dopaminergic System in Tau Pathology

In forms of neurodegenerative diseases that include symptoms of parkinsonism, a severe deficiency in dopamine signaling underlies motor dysfunction [65,66]. Of particular relevance here are mutations in the tau gene, which cause frontotemporal dementia with parkinsonism linked to chromosome 17 (FTDP-17) in 30% of patients [67]. The P301L/S mutations, which are strongly linked with FTDP-17, reduce the binding affinity between tau and microtubules [68]. Tau mutated at P301 is therefore prone to aggregation and, in L66, was expressed under the control of the neuron-specific regulatory element Thy-1. The underlying mechanisms for motor impairment in this model are not yet understood [11,69]. Methylthioninium chloride (MTC) and hydromethylthionine mesylate (HMTM) reverse the motor deficits in L66 mice [11,54]. These drugs also mitigate deficiencies in glutamate signaling in crude whole-brain synaptosomes [40] and normalize cholinergic activity [45,70,71] while at the same time reducing tau levels [69]. Tau expression in dopaminergic neurons/regions and their functional consequences, however, remained elusive so far.

A key finding in this study is that 9-month-old L66-Homo mice were motorically impaired, while L66-Het mice of the same age showed no motor impairment, performing similarly to age-matched NMRI controls. Furthermore, compared to L66-Het mice, L66-Homo presented with (1) a more pronounced decline in dopaminergic markers in the substantia nigra and striatum, and (2) a greater level of tau deposits in the cerebral cortex and striatum. This raises the question of whether these two discoveries can explain the observed motor disorders, and which specific alterations result from overexpression of mutant tau.

In L66-Homo mice, the approximately 40% reduction in the number of TH-immunoreactive dopaminergic cells was significant, whereas the magnitude of the reduction in L66-Het mice was approximately half of that observed in L66-Homo mice, consistent with a gene dosage effect. Despite this reduction in TH immunoreactivity, L66-Het mice did not exhibit motor deficits in either the accelerated or constant-speed RotaRod protocols (Figure 2 and Figure 3) or in the inverted grid test (Figure 4). Thus, the degree of dopaminergic alteration observed in L66-Het mice was not associated with detectable motor impairment under the conditions tested.

One possible interpretation is that the dopaminergic alterations in L66-Het mice remain below a functional threshold required to produce overt motor deficits. This interpretation is broadly compatible with observations from Parkinson’s disease, in which substantial nigrostriatal dopaminergic degeneration can occur before the manifestation of motor symptoms, with estimates of approximately 50–70% neuronal loss in the substantia nigra and ~70–80% reductions in striatal dopamine levels at symptom onset [65,72,73]. However, these values derive from human Parkinson’s disease and should not be considered a defined threshold for the L66 model. Rather, they provide a possible conceptual framework for interpreting the absence of motor deficits in L66-Het mice. Importantly, our dataset does not allow us to establish a specific threshold or to determine whether the behavioral phenotype in L66-Homo mice is exclusively attributable to reduced dopaminergic tone. Although some markers showed a trend toward greater loss in L66-Homo mice, the differences from L66-Het mice were often not statistically significant. Further studies with larger sample sizes and a broader range of dopaminergic deficits would be required to directly test whether a quantitative relationship or threshold exists between dopaminergic loss and motor impairment in this model.

The observed changes in VMAT-2-stained neurons, however, do suggest that the deficits observed in RotaRod and inverted grid are indeed due to alterations in dopaminergic activity. Both wild-type and L66-Het mice showed high levels of VMAT-2, but in L66-Homo mice levels were significantly lowered and associated with a motor phenotype. Such a relationship has recently been described for an experimental model of Parkinson’s disease by Lyu and colleagues [74]. Animals treated with MPTP failed in the RotaRod and the levels of TH and VMAT-2 were a tenth of those in wild-type controls. In their hands, repeated transcranial magnetic resonance exposure not only recovered the motor phenotype, but it also raised TH and VMAT-2 levels to 50–80% of wild-type levels. This clearly suggests that a reduction of ~20% of TH neurons in the SNpc, as observed in L66-Het mice, is insufficient for induction of deficits in the RotaRod. In this study we were able to show that the inverted grid test shows the same sensitivity as the RotaRod and provides verification of our previous work [29].

The motor phenotype of L66-Homo mice does not differ substantially from that of other lines carrying the P301S mutation. Similar to other P301S models, such as Thy1 hTau P301S and PS19 [75,76], L66 mice develop extensive tau pathology and early, progressive motor deficits affecting their performance in several behavioral tests, including the RotaRod, balance beam and normal gait using the Catwalk [11]. In P301S lines, motor deficits emerge at approximately four months of age, as demonstrated by RotaRod and grasping tests, and progress to muscle weakness and reduced mobility. By nine months of age, P301S mice exhibit a significant increase in locomotor activity, indicated by hyperactivity and behavioral disinhibition. This progression is more pronounced than in the majority of other tau models [77,78,79]. Many P301S transgenic models utilize strong neuronal promoters, such as Thy1 or PrP, which drive high transgene expression in motor system neurons. P301L models, which are characterized by a divergent mechanism of tau protein aggregation and a slower but stable accumulation of pathological tau compared to P301S mice, are frequently used for comparative analyses with P301S lines [80,81]. Evidence of strong tau pathology has been observed in the cortex and hippocampus of these mice. Some P301L lines have been observed to exhibit gait abnormalities or RotaRod deficits; however, the motor impairments observed in these lines are generally milder and progress more slowly than in P301S models [82,83]. Other tau models that do not involve P301 mutations, including wild-type human tau overexpression and mixed 3R/4R tau lines, typically exhibit either an absence of or only mild motor deficits, especially at early ages. In the event of motor changes in these models, they are typically characterized by a late onset and subtlety, with cognitive and synaptic deficits being the predominant features. Research findings emphasize the pivotal function of striatal tau pathology in the mediation of motor symptoms, frequently resulting in the manifestation of parkinsonian-like phenotypes. Recent studies have demonstrated that local modulation of tau isoforms can enhance the 3R:4R ratio in the striatum, thereby restoring motor coordination deficits [84]. TAU58/2 mice, which carry the P301S mutation under a different promoter (mThy1.2), also show altered motor behavior, but these changes are less well characterized than those reported for PS19 and Thy1 P301S mice. The literature has focused more on cognitive and synaptic dysfunction than on motor outcomes; however, early-onset progressive behavioral deficits have been observed [59,85,86].

### 4.3. The Relationship Between Tau Accumulation and the Dopaminergic System

Reasons for the reduced number of TH-immunoreactive (ir) neurons, which are greatest in L66-Homo and milder in L66-Het, remain unclear. We originally proposed that there would be a strong correlation between tau expression and dopaminergic markers, but this was not corroborated by correlation analyses (see Supplementary Information Table S2). Indeed, both motor cortex and striatum presented with expression of human tau, implicating tau toxicity in the diminished labelling of TH in cells and fibers. But the reduction of TH in the substantia nigra was apparently not due to tau expression in L66 mice as recorded by 7/51 or S-1D12 (but may be sensitive to antibodies against other epitopes). The absence of tau-positive immunoreactivity was confirmed with two highly specific antibodies, which provided strong labelling in neighboring regions such as the red nucleus or lateral geniculate nucleus. However, given the strong tau labelling in striatal aspects of the basal ganglia, which coincided with diminished TH immunoreactivity, some retrograde degeneration of nigro-striatal efferents cannot be excluded. Others who reported dopaminergic alterations in tau-overexpressing mice have also noticed a relative paucity of tau-ir in areas projecting to the striatum [14]. Firstly, it should be noted that the reduction in the number of TH-ir neurons in the SNpc in L66-Het mice was accompanied by only a slight increase in anti-tau staining in the striatum compared with the control group. This increase is less pronounced than that observed in L66-Homo mice, as was the loss of TH-ir neurons in the SNpc. Consequently, the variation in anti-tau staining intensity within the striatum between the control group and L66-Het mice is deemed to be statistically insignificant. This is also the case for the discrepancy in the number of TH-ir neurons in the SNpc between control mice and L66-Het mice. The observed differences in both the control group and the L66-Homo mice are statistically significant. The observed association between the loss of TH-ir neurons in the SNpc and the intensity of anti-tau staining in the striatum suggests a potential role for striatal tauopathy in the reduction of TH-ir neurons in the SN.

An alternative explanation may be based on the reciprocal connectivity between striatum and substantia nigra. It has been established that striatal efferents arising from medium spiny neurons can, via both direct and indirect pathways, modulate activity of dopaminergic cells in the substantia nigra. From the direct pathway, striatal GABAergic projections terminate in the substantia nigra [87]. In monkeys, this projection is physiologically relevant as dopamine outflow from the globus pallidus is regulated by oscillatory activity in the 3–15 Hz range in the substantia nigra. It can be recovered by D1/D5 receptor activation in the globus pallidus in MPTP monkeys [88], providing compelling functional support for this GABAergic connection, also revealed in the rodents [89,90,91,92,93]. In addition, both the subthalamic nucleus and globus pallidus project excitatory efferents to the substantia nigra [94]. It appears that these nigral endings are also under the control of nigrostriatal collaterals releasing dopamine. We thus envisage that the reduced neural activity in the striatum due to tau overexpression may lead to secondary effects in substantia nigra neurons.

Although we did not explore in detail which cell types are affected in the striatum, it is likely that both fast-spiking GABAergic and cholinergic interneurons are affected by the reduction in TH in L66-Homo mice. Simultaneous degeneration of both cell types is correlated with the death of substantia nigra dopaminergic neurons. This has been attributed to a lowering of trophic support via glial cell-line derived neurotrophic factor (GDNF), which is normally produced by striatal efferents to the substantia nigra and would explain the loss of dopaminergic cells in the SNpc in terms of molecular mechanisms [95]. Interestingly, despite the well-documented deficits in dopaminergic markers within the substantia nigra, striatum, and motor cortex of L66 homozygote animals, tauopathy was not detected in the substantia nigra. This unexpected dissociation suggests that the observed motor impairments in these mice may be more closely associated with pathological changes or disrupted functional regulation within higher-order motor structures, particularly the motor cortex, rather than direct degeneration of the dopaminergic neurons in the substantia nigra.

### 4.4. The Role of Descending Cortico-Striatal Glutamatergic Projection

By contrast to the substantia nigra, which was free of tau labelling, strong expression of tau was detected in the cortex of L66-Homo mice. All three tau antibodies provided similar findings in L66 and these results are in line with results from other P301L/S transgenic mice [52,96]. Importantly, a similar model to our L66 was developed in the laboratory of Virginia Lee and John Trojanowski [97] and tangle-like inclusions were confirmed in the cortex concomitant with neurofilament light chain labelling, confirming neurodegeneration. Frank cell loss occurred only in animals at 12 months or older. This pattern was also observed in L66-Homo mice, which showed clear morphological changes consistent with neuronal injury in cresyl violet-stained cortical sections, despite the absence of detectable neuronal loss in NeuN staining. Whether older L66 Homo mice would, like PS19 mice, also present with cortical thinning and cell loss remains elusive. Although glutaminase does not exclusively stain neurons, but is also expressed in glia, the glutaminase staining seems to suggest that, despite the apparent absence of overt neuronal loss, cortical functionality may be compromised. Such functional alterations likely contribute to the changes in glutamatergic signaling observed within the basal ganglia circuitry. In parallel, other experimental tauopathy models have revealed alterations in glutamate homeostasis and metabolism, including changes in glutamate turnover, release, and clearance [98,99,100,101]. Together, these findings suggest that dopaminergic dysfunction and altered glutamatergic metabolism may represent interconnected components of circuit dysfunction in tauopathy.

The increased number of shrunken neurons in the secondary motor cortex in L66-Homo mice strongly suggests a progressive atrophy in this line. However, this did not occur uniformly across the cortex but seemed more prominent in M2 than in M1 areas. Due to the low number of samples, the data were not significant; a more widespread investigation of the cortex would clarify this issue. Although not examined directly, this increased degenerative profile in L66 mice would suggest disease-relevant synaptic modifications [102], thereby lowering the glutamatergic drive from motor cortex to basal ganglia and may provide another source for lower TH levels in the striatum/substantia nigra. Our glutaminase staining appears to corroborate the notion that there is a lack of cell loss but still a loss of cortical functionality, which has dramatic consequences on the striatum. All the above interpretations require qualification, as the data are from female mice only and require confirmation in male subjects.

## 5. Conclusions

The distinct motor impairment in 9-month-old female L66-Homo, which is absent in L66-Het mice, is accompanied by a greater loss of dopaminergic staining in SNpc and strong tau expression in the striatum and motor cortex. Since overexpression of human tau is unlikely to have caused cell loss in the cortex and SNpc of L66-Homo mice, but is present in the striatum, we suggest this phenotype results from tau-related deficiencies in functional outputs of the dopaminergic system. Such functional changes, observed at least in female mice, include striatal and SNpc dysfunction and are markedly exacerbated by modifications in the motor cortex. Further studies are needed to determine whether these findings also apply to male mice.

## Figures and Tables

**Table 1:** Primary and secondary antibodies used for immunohistochemistry.

Primary Antibody	Supplier	Dilution	Secondary Antibody	Supplier	Dilution
Anti-TH (Cat. No AB152)	Merck	1:1000	Goat anti-rabbit (Cat. No AP307P)	Merck	1:200
Anti-VMAT-2 (Cat. No 20042)	Immunostar	1:500	Goat anti-rabbit (Cat. No AP307P)	Merck	1:200
7/51 anti-tau (Epitope tau343-355)	TauRx Therapeutics Ltd.	1:500	Goat anti-mouse (Cat. No AP124P)	Merck	1:200
AT8 anti-tau (Cat. No MN1020) (Epitope pSer202, pThr205)	Thermofisher Scientific	1:200	Goat anti-mouse (Cat. No AP124P)	Merck	1:200
S-1D12 anti-tau (Epitope tau337-355)	TauRx Therapeutics Ltd.	1:1000	Goat anti-mouse (Cat. No AP124P)	Merck	1:200
NeuN (Cat. No MAB377)	Merck	1:1000	Goat anti-mouse (Cat. No AP124P)	Merck	1:200
Anti-glutaminase (Cat. No PA5-35365)	Thermofisher Scientific	1:100	Goat anti-rabbit (Cat. No AP307P)	Merck	1:200

## Data Availability

The raw data supporting the conclusions of this article will be made available by the authors on request.

## Supplementary Materials

The following supporting information can be downloaded at https://www.mdpi.com/article/10.3390/cells15201908/s1, Table S1: The parameters of movement characteristic, along with their definitions, measured in the Inverted Horizontal Grid test; Figure S1: Mean body weights recorded prior to first behavioral testing (accelerated RotaRod); Figure S2: Schematic illustration of the organization of serial brain sections obtained from frozen tissue; Figure S3: Schematic illustration of the organization of serial brain sections obtained from paraffin-embedded tissue; Table S2: Regression analysis of the associations between dopaminergic markers and tau protein levels measured using different antibodies in L66 Homo mice.

## Abbreviations

%Area	Percentage area covered with staining.
7/51	Phosphorylation-independent tau antibody, labelling mutant human tau and native murine tau (epitope 350–368).
Ab	Antibody.
ACC	Accelerated rod rotation.
ANOVA	Factorial two-way analysis of variance.
AT8	Phospho-tau (Ser202, Thr205) monoclonal antibody.
BSA	Bovine serum albumin.
CBD	Corticobasal syndrome.
Cx	Cortex.
D1	Dopamine receptor type 1.
D2	Dopamine receptor type 2.
D5	Dopamine receptor type 5.
DAB	3,3′-Diaminobenzidine tetrahydrochloride.
DePeX	Neutral solution of polystyrene and plasticizers in xylene, mounting medium.
DMSO	Dimethyl sulfoxide.
Exp	Experiment.
FIX	Constant rod rotation speed.
FL	Front left paw.
FR	Front right paw.
FTD	Frontotemporal dementia.
FTDP-17	Frontotemporal dementia with parkinsonism linked to chromosome 17.
GABA	Gamma-aminobutyric acid.
GDNF	Glial cell-line derived neurotrophic factor.
HL	Hind left paw.
HMTM	Hydromethylthionine mesylate.
HR	Hind right paw.
HRP	Horseradish peroxidase.
ir	Immunoreactive.
ITI	Inter-trial interval.
L1	Line 1 transgenic NMRI- derived Alzheimer disease-like tauopathy mouse model.
L66	Line 66 transgenic NMRI-derived frontotemporal dementia like tauopathy mouse model.
L66-Het	Line 66 heterozygous mice (L66^+/−^).
L66-Homo	Line 66 homozygous mice (L66^+/+^).
LTP	Long-term potentiation.
M1	Primary motor cortex.
M2-	Secondary motor cortex.
MAPT	Microtubule-associated protein tau.
MATLAB	MathWorks, Massachusetts.
MPEG	Moving Picture Experts Group.
MPTP	1-Methyl-4-phenyl-1,2,3,6-tetrahydropyridine.
MTC-	Methylthioninium chloride.
NeuN	Neuronal marker express in the nuclei and cytoplasm of neurons.
NFT	Neurofibrillary tangle.
NGS	Normal goat serum.
NMRI	Naval Medical Research Institute mouse model, wild-type mice.
NRS	Normal rabbit serum.
NS	Not significant.
PBS	Phosphate-buffered saline.
PD	Parkinson’s disease
Thy-1	Neuron-specific promoter.
PSP	Progressive supranuclear palsy.
ROI	Relative optical intensity.
rpm	Rotation per minute.
RT	Room temperature.
S-1D12	High-affinity chimeric monoclonal antibody targeting the core microtubule-binding region of tau protein (epitope 337–355).
SD	Standard deviation
SNpc	Substantia nigra pars compacta.
St-	Striatum.
*t₍b₎*	The timestamps marking the onset.
*t₍e₎*	Termination of the hold.
TH	Tyrosine hydroxylase.
Thy-1	Neuron-specific Thy-1 promoter.
VMAT-2	Vesicular monoamine transporter type 2.
WT	Wild-type mice.
