# Supplementary material for: Motor Deficits in a Mouse Model of Frontotemporal Dementia: Cortical Tau Pathology and Reduced Nigrostriatal Dopaminergic Markers
Source: Cells. 2026 Oct 20;15(20):1908. doi: 10.3390/cells15201908 (PMC13702886; doi:10.3390/cells15201908)
Supplement: Supplementary file 1 [file cells-15-01908-s001.zip › cells-4460269-supplementary.pdf]

Supplementary information

Table S1. The parameters of movement characteristic, along with their definitions, measured in the Inverted Horizontal Grid test.

**Parameters of movement characteristic**	**Definition**	**Normalization**
Total Number of Steps	Number of all Steps during the trial	Yes
Overall Duration of Locomotion [s]	Sum of durations of all Steps during the trial	Yes
Cumulative Step Distance [mm]	Sum of length of all Steps during trial	Yes
Mean Step Duration [s]	Total Steps Duration/Total Number of Steps	No
Mean Step Length [mm]	Total Steps Distance/ Total Number of Steps	No
Mean Step Velocity [mm/s]	Total Steps Distance/Total Steps Duration	No

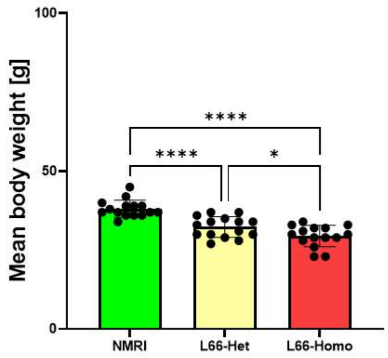

Figure S1. Mean body weights recorded prior to first behavioral testing (accelerated RotaRod) of wild-type NMRI (n=15), L66-Het (n=15) and L66-Homo (n=15) mice.

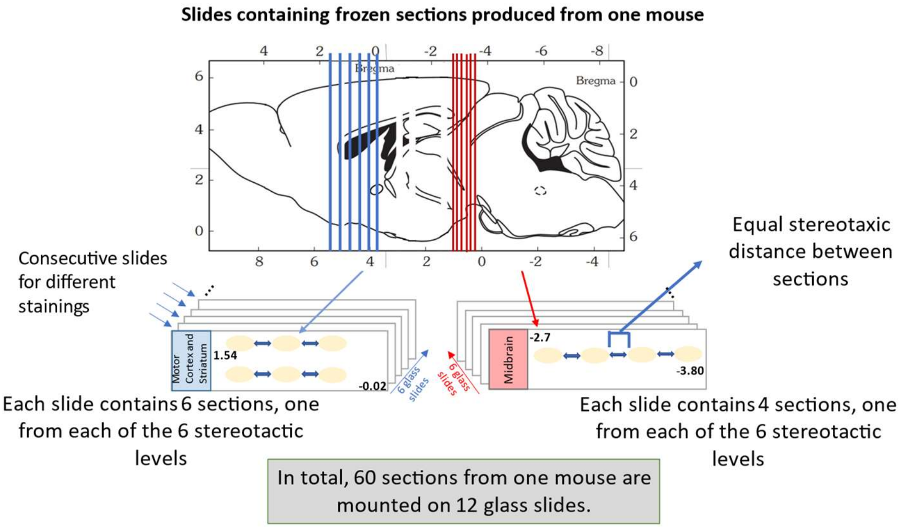

Figure S2: Schematic illustration of the organisation of serial brain sections (40 μm thick) obtained from frozen tissue from a single mouse brain and arranged according to stereotaxic coordinates. For the motor cortex and striatum (blue lines), sections were collected from 1.54 to -0.02 mm anteroposterios relative to Bregma; midbrain sections (red lines) were collected from -2.70 to -3.80 mm posterios to Bregma. In total, 60 sections from a single brain were distributed across 12 microscope slides to enable subsequent immunohistochemical analysis of multiple protein targets.

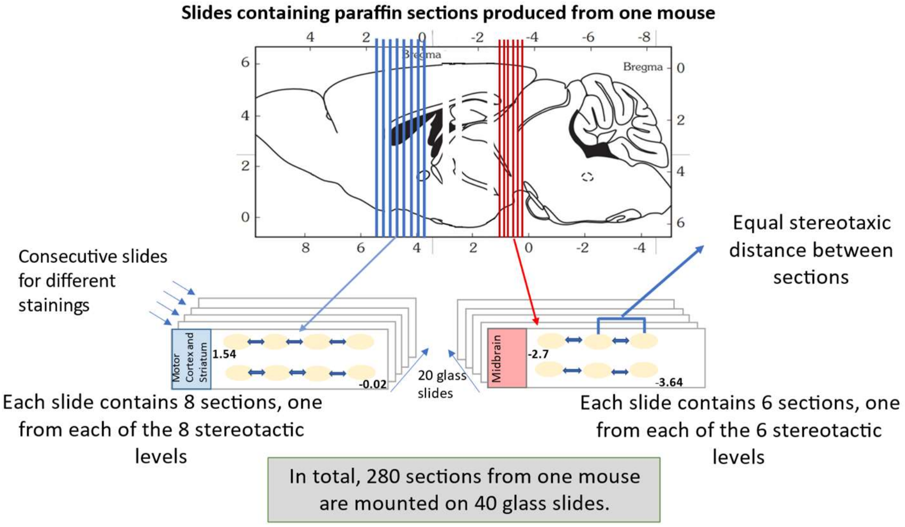

Figure S3: Schematic illustration of the organisation of serial brain sections (6 μm thick) obtained from paraffin-embedded tissue from a single mouse brain and arranged according to stereotaxic coordinates. For the motor cortex and striatum (blue lines), sections were collected from 1.54 to -0.02 mm anteroposterios relative to Bregma; midbrain sections (red lines) were collected from -2.70 to -3.80 mm posterios to Bregma. In total, 280 sections from a single brain were distributed across 40 microscope slides to enable subsequent immunohistochemical analysis of multiple protein targets.

Table S2. Regression analysis of the associations between dopaminergic markers and tau protein levels measured using different antibodies in L66 Homo mice.

Analysis for SNpc vs. Striatum

**Mice L66 Homo**	**SNpc TH vs. Striatum AT8**	**SNpc TH vs. Striatum 7/51**	**SNpc VMAT-2 vs. Striatum AT8**	**SNpc VMAT-2 vs. Striatum 7/51**
Best-fit values				
Slope	-0,01371	0,009907	-0,07677	0,1192
Y-intercept	36,95	56,65	43,23	41,1
X-intercept	2695	-5718	563	-344,7
1/slope	-72,94	100,9	-13,03	8,387
Std. Error				
Slope	0,04034	0,05415	0,159	0,1016
Y-intercept	7,672	8,991	19,15	13,39
95% Confidence Intervals				
Slope	-0,1257 to 0,09830	-0,1624 to 0,1823	-0,5828 to 0,4292	-0,1629 to 0,4014
Y-intercept	15,65 to 58,25	28,04 to 85,27	-17,71 to 104,2	3,914 to 78,28
X-intercept	452,1 to +infinity	-infinity to -158,4	176,0 to +infinity	-infinity to -9,879
Goodness of Fit				
R squared	0,02807	0,01103	0,07212	0,256
Sy.x	5,225	6,813	5,588	6,505
Is slope significantly non-zero?				
F	0,1155	0,03347	0,2332	1,377
DFn, DFd	1, 4	1, 3	1, 3	1, 4
P value	0,7511	0,8665	0,6622	0,3058
Deviation from zero?	**Not Significant**	**Not Significant**	**Not Significant**	**Not Significant**
Equation	Y = -0,01371*X + 36,95	Y = 0,009907*X + 56,65	Y = -0,07677*X + 43,23	Y = 0,1192*X + 41,10
Data				
Number of X values	6	5	5	6
Maximum number of Y replicates	1	1	1	1

Total number of values	6	5	5	6
Number of missing values	0	0	0	0

Analysis within the striatum

**Mice L66Homo**	**Striatum TH vs. Striatum AT8**	**Striatum TH vs. Striatum 7/51**	**Striatum VMAT-2 vs. Striatum AT8**	**Striatum VMAT-2 vs. Striatum 7/51**
Best-fit values				
Slope	0,476	-1,343	0,8736	-1,167
Y-intercept	9,441	125,4	-17,65	121,3
X-intercept	-19,84	93,4	20,21	103,9
1/slope	2,101	-0,7447	1,145	-0,8572
Std. Error				
Slope	0,8974	0,8894	0,6416	0,3859
Y-intercept	46,56	45,71	37,2	21,67
95% Confidence Intervals				
Slope	-2,380 to 3,332	-3,812 to 1,127	-1,887 to 3,634	-2,395 to 0,06137
Y-intercept	-138,7 to 157,6	-1,500 to 252,3	-177,7 to 142,4	52,30 to 190,2
X-intercept	-infinity to 41,90	65,98 to +infinity	-infinity to 49,32	79,06 to +infinity
Goodness of Fit				
R squared	0,08573	0,363	0,481	0,7529
Sy.x	5,593	6,019	4,393	4,257
Is slope significantly non-zero?				
F	0,2813	2,279	1,854	9,141
DFn, DFd	1, 3	1, 4	1, 2	1, 3
P value	0,6326	0,2056	0,3065	0,0566
Deviation from zero?	**Not Significant**	**Not Significant**	**Not Significant**	**Not Significant**
Equation	Y = 0,4760*X + 9,441	Y = -1,343*X + 125,4	Y = 0,8736*X - 17,65	Y = -1,167*X + 121,3
Data				
Number of X values	5	6	4	5

Maximum number of Y replicates	1	1	1	1
Total number of values	5	6	4	5
Number of missing values	0	0	0	0
